# A nonsurgical brain implant enabled through a cell–electronics hybrid for focal neuromodulation

**DOI:** 10.1038/s41587-025-02809-3

**Published:** 2025-11-05

**Authors:** Shubham Yadav, Ray X. Lee, Shivam N. Kajale, Baju Joy, Monochura Saha, Preet Patel, Loey Bull, Sarah Cao, Samir Mitragotri, David Bono, Deblina Sarkar

**Affiliations:** 1 MIT Media Laboratory, Massachusetts Institute of Technology, Cambridge, MA USA; 2 Department of Neuroscience, Wellesley College, Wellesley, MA USA; 3 Department of Chemical Engineering, Massachusetts Institute of Technology, Cambridge, MA USA; 4 John A Paulson School of Engineering and Applied Sciences, Harvard University, Allston, MA USA; 5 Wyss Institute for Biologically Inspired Engineering, Boston, MA USA; 6 Department of Materials Science and Engineering, Massachusetts Institute of Technology, Cambridge, MA USA

**Keywords:** Biotechnology, Biomedical engineering

## Abstract

Bioelectronic implants for brain stimulation are used to treat brain disorders but require invasive surgery. To provide a noninvasive alternative, we report nonsurgical implants consisting of immune cell–electronics hybrids, an approach we call Circulatronics. The devices can be delivered intravenously and traffic autonomously to regions of inflammation in the brain, where they implant and enable neuromodulation, circumventing the need for surgery. To achieve suitable electronics, we designed and built subcellular-sized, wireless, photovoltaic electronic devices that harvest optical energy with high power conversion efficiency. In mice, we demonstrate nonsurgical implantation in an inflamed brain region, as an example of therapeutic target for several neural diseases, by employing monocytes as cells, covalently attaching them to the subcellular-sized, wireless, photovoltaic electronic devices and administering the resulting hybrids intravenously. We also demonstrate neural stimulation with 30-µm precision around the inflamed region. Thus, by fusing electronic functionality with the biological transport and targeting capabilities of living cells, this technology can form the foundation for autonomously implanting bioelectronics.

## Abstract (web summary)

Photovoltaic devices attached to immune cells travel through the blood to inflamed brain regions.

## Main

Electronic devices implanted in the body provide powerful tools for diagnosis, therapeutics and research^1–4^. For example, bioelectronic implants for brain stimulation have provided biological insights and have proved effective for treating many brain diseases^1^. However, placing a medical implant inside the brain typically requires invasive intracranial surgery, with associated pain and tissue damage along with risks of infection, ischemia, psychological distress, morbidity and mortality^5^. Even endovascular electrodes^6–8^, although not requiring intracranial access, still need endovascular surgery with its associated risks and complications. Moreover, they cannot achieve submillimeter spatial targeting precision and are unable to access most brain regions^6–8^. While attempts have been made to explore intravenous (i.v.) injection routes^9^, these have led to nonspecific stimulation of large brain regions without focality. On the other hand, existing noninvasive brain stimulation technologies, such as transcranial magnetic stimulation and transcranial direct current stimulation, lack the necessary spatio-temporal resolution^10^.

**Fig. 1:**
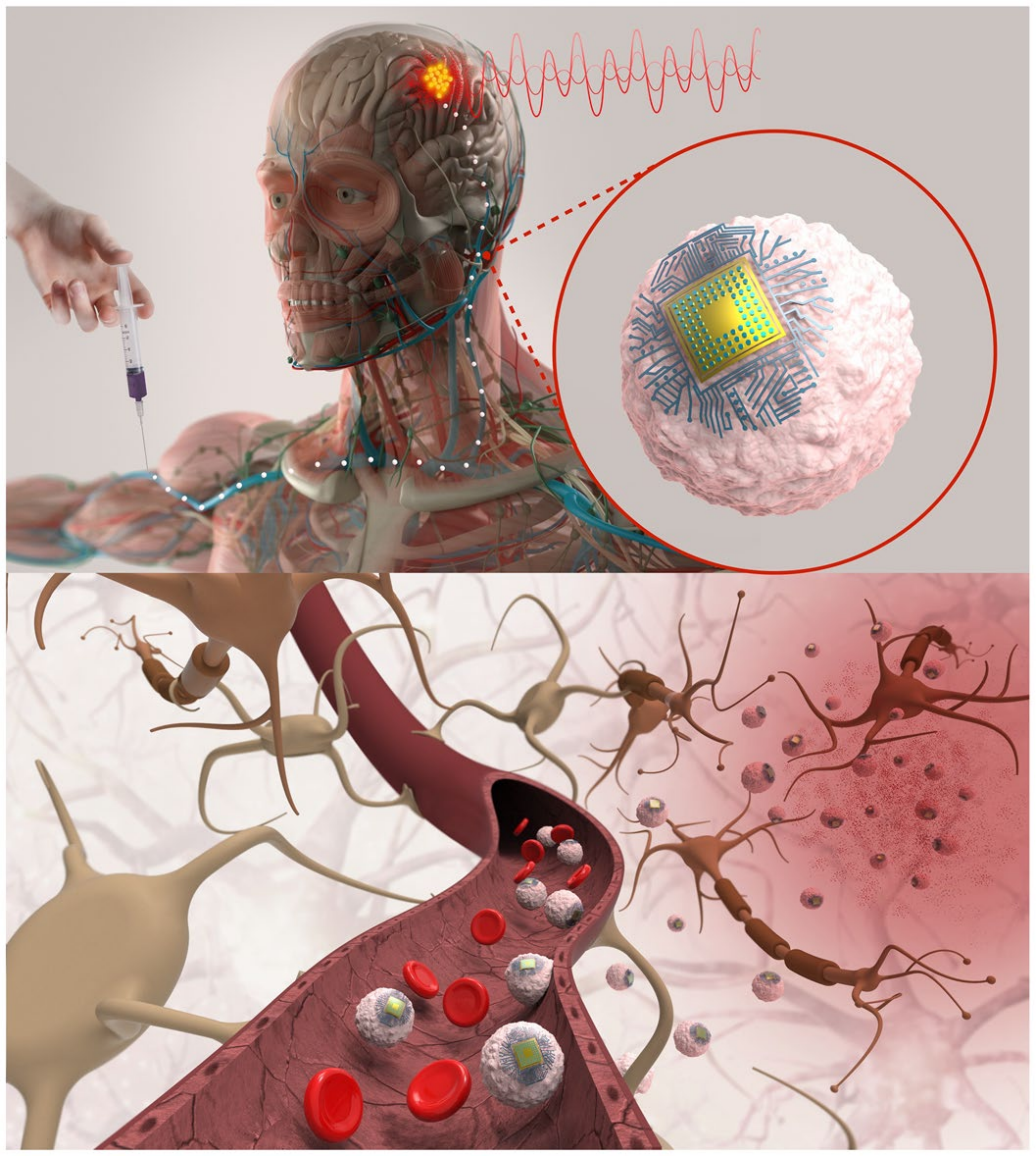
Circulatronics. Schematic diagram illustrating the concept of Circulatronics. Credit: Pablo Penso; human anatomy image from Shutterstock.

Here we have developed bioelectronic devices that, after i.v. injection, are trafficked through the circulatory system and implant autonomously in brain regions of inflammation. We also demonstrate that they enable wirelessly controlled focal stimulation of deep brain regions such as ventrolateral thalamic nucleus in the rodent brain providing a nonsurgical brain implant for focal neuromodulation that takes advantage of immune cells’ natural trafficking to sites of inflammation. We name electronics that circulate through the vasculature ‘Circulatronics’ (Fig. 1). Realization of the Circulatronics brain stimulator requires overcoming several hurdles: (1) development of efficient wireless free-floating electronic devices that are miniaturized to fit inside the vasculature, (2) circulation of these devices without being eliminated from the bloodstream and (3) recognition of and self-implantation in desired brain regions. To overcome these challenges, we built wireless optical energy harvesting electronic devices that are subcellular sized and self-standing with high efficiency (to achieve point (1)) and created hybrids with living immune cells (to achieve points (2) and (3)). We demonstrate this technology for brain regions of inflammation, an important therapeutic target for many neurologic diseases, including Alzheimer’s disease^11,12^, multiple sclerosis^13^, ischemic stroke^14^, brain tumor^15^, neuropathic pain^16–18^, spinal cord^19^ and peripheral nerve injury^20^, whose treatment may benefit from electrical modulation targeted at the inflamed region^11–20^. We describe the electronic device design and fabrication, the creation of cell–electronics hybrids, the nonsurgical focal brain stimulation and biocompatibility studies.

## Results

### Subcellular-sized free-floating wireless electronic devices

**Fig. 2:**
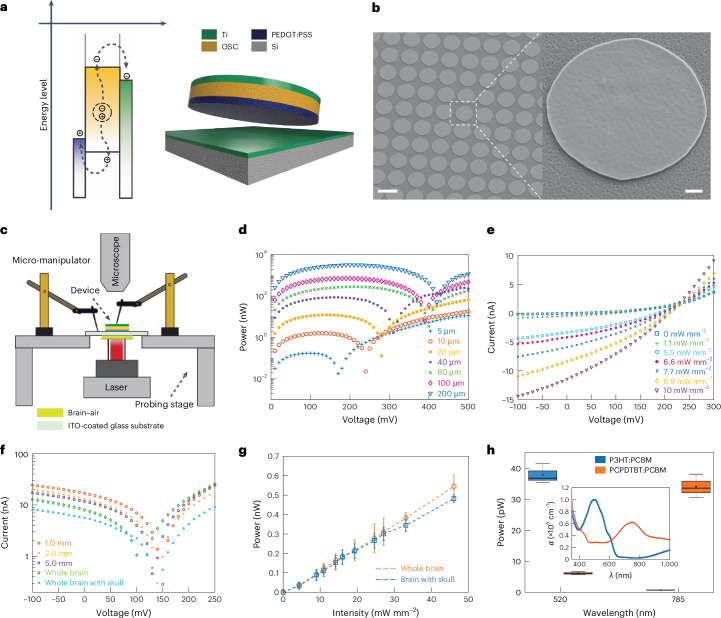
Characterization of subcellular-sized electronics with optical remote control. **a**, Schematic energy diagram illustrating the working principle of the photovoltaic devices (three-layered device based on organic polymers) and the final device structure developed. OSC, organic semiconductor. **b**, SEM images of the SWEDs fabricated at wafer scale. SWED structure, PEDOT:PSS|P3HT:PCBM|Ti. Scale bar, 10 µm (inset, 1 µm), *n* = 5. **c**, Schematic diagram of the setup used for in-air and ex vivo measurements. The devices were illuminated from the bottom with a laser. For ex vivo measurements, brain tissue or whole brain with the skull was placed on a no. 1.5 cover slip (while keeping the tissue wet in PBS) and in close contact with the devices. The microscope was used to assist with alignment while probing the devices from the top using the three-stage micromanipulators. The probes were connected to a potentiostat and the laser was operated in continuous-wave mode to obtain the current–voltage (*I*–*V*) curves. In-air measurements were as follows: **d**, Representative plot showing the power generated by different-sized devices as a function of applied voltage. Device structure, PEDOT:PSS|P3HT:PCBM|Ti, Intensity 10 mW mm^−^^2^ incident on the devices, *n* > 5 devices. Legend, device diameter. **e**, Representative *I*–*V* characteristics of a SWED (10 µm in diameter) for varied light intensities incident on the SWED. SWED structure, PEDOT:PSS|P3HT:PCBM|Ti, *n* > 5 devices. A 520-nm wavelength laser was used as the light source for **d** and **e**. Ex vivo measurements: **f**, Representative *I*–*V* plot for the SWEDs (10 µm in diameter) with light passing through different thicknesses of brain slices or whole brain (6.1 ± 0.3 mm) or whole brain with skull (6.5 ± 0.3 mm). The transmittance of NIR penetration through the brain tissue was measured and is presented in Supplementary Fig. 9 (Methods section ‘NIR light transmittance measurements’). SWED structure, PEDOT:PSS|PCPDTBT:PCBM|Ti, *n* = 3 devices. Laser, 792 nm; intensity, 24.6 mW mm^−^^2^ incident on the bottom surface of the brain. **g**, Maximum power generated by the SWEDs (10 µm in diameter) with light passing through the whole brain without the skull (and the whole brain with an intact skull) at different light intensities (792 nm) incident on the bottom surface of the brain. SWED structure, PEDOT:PSS|PCPDTBT:PCBM|Ti, values represent median ± standard deviation (s.d.) (*n* = 5 devices). **h**, Box plot showing the multiplexing effect using the two SWED structures (10 µm in diameter) we have developed: PEDOT:PSS|PCPDTBT:PCBM|Ti and PEDOT:PSS|P3HT:PCBM|Ti. The plot shows the mean (circle within the box), median (horizontal line within the box), lower and upper quartile (delineated by the box), whiskers extending to the most extreme data points within 1.5× IQR from the quartiles, with minima and maxima beyond this range shown as individual outlier points (*n* = 3 devices per condition). The inset shows the absorption coefficients for the two SWED structures indicating their orthogonal absorption coefficients at the operating wavelengths. Labels, *α* (absorption coefficient, ×10^5^ cm^−1^) and *λ* (wavelength, nm). The measurements were done with light passing through a 0.5-mm-thick brain tissue slice. Intensities used for this measurement were 10 mW mm^−^^2^ (520 nm) and 2 mW mm^−^^2^ (785 nm) incident on the bottom surface of the brain slice. Source data

**Extended Data Fig. 1:**
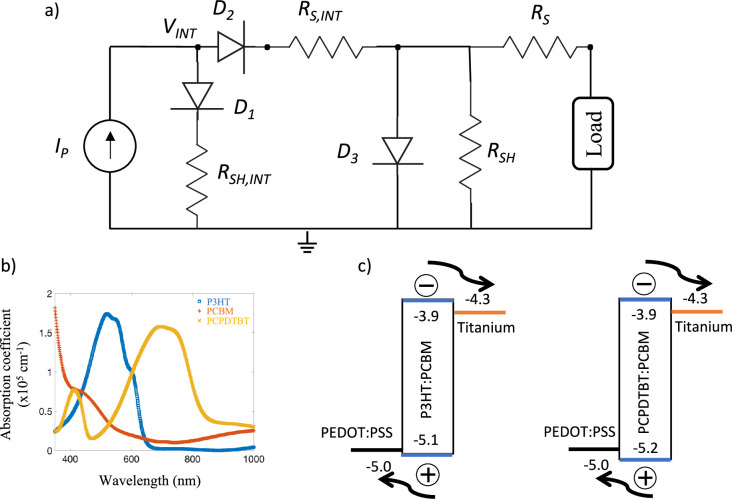
Three-diode electrical model, polymer characterization and band diagram for the developed photovoltaic devices. (**a**) Equivalent circuit diagram used for modeling organic photovoltaic devices^50^. This model differs from the standard model for silicon photodiodes in regard to generation of excitons (bound electron-hole pairs) instead of free electron-hole pairs and as such needs additional elements to model the transition of excitons to electron-hole pairs. Here, *I*_*P*_ represents the number of polaron pairs (spatially separated bounded electron-hole pairs) generated following dissociation of excitons after diffusing to the nearest donor/acceptor interface and is assumed to be constant for a given optical intensity. The loss of polarons is modeled using *R*_*SH,INT*_ (polaron-pair recombination) and *R*_*S,INT*_ (resistance experienced by polarons after forming free charges and reaching to the electrodes). *D*_*1*_ and *D*_*2*_ are modeled as ideal diodes and behave like short-circuit (open-circuit) when under forward (reverse) bias. *D*_*3*_, *R*_*SH*_ and *R*_*S*_ are used to model the current-voltage characteristics of the device in the absence of light. (**b**) Absorption coefficients for the various organic semiconducting polymers used in this work - P3HT, PCPDTBT and PCBM. From the plot, it can be inferred that P3HT has a peak absorption around 520 nm and PCPDTBT has a peak absorption in the near-infrared (750 nm) region. (**c**) Energy band diagrams are shown for PEDOT:PSS|P3HT:PCBM|Ti (left) and PEDOT:PSS|PCPDTBT:PCBM|Ti (right). P3HT:PCBM and PCPDTBT:PCBM (binary blends) creates bulk heterojunctions that ensures that all the excitons generated by the incident photons get dissociated into individual electron-hole pairs within their diffusion lengths. Since, there is a built-in electric field created due to the differences in the work function of the anode (PEDOT:PSS) and cathode (Ti), the newly created electrons and holes are drawn efficiently towards the cathode and the anode side, respectively, minimizing recombination in the active layer (binary blend). Source data

**Extended Data Fig. 2:**
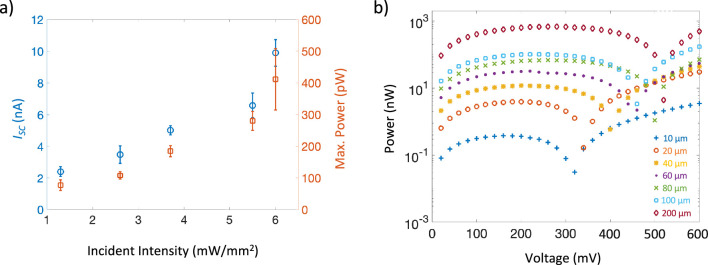
PCPDTBT device performance. (**a**) Short circuit current and maximum power generated by subcellular sized devices (10 µm in diameter) for varied optical intensities. Device structure – PEDOT:PSS|PCPDTBT:PCBM|Ti, laser – 785 nm, values represent median ± SD (n=3 devices). (**b**) Power-voltage plot (semi log) for differently sized devices. Plot shows that the power generated by these devices scale up with increase in device size. Device structure – PEDOT:PSS|PCPDTBT:PCBM|Ti, intensity – 6 mW/mm^2^. Legends are corresponding to the device diameter. These are representative data from measurements of at least 5 independent devices for each size. Source data

**Extended Data Fig. 3:**
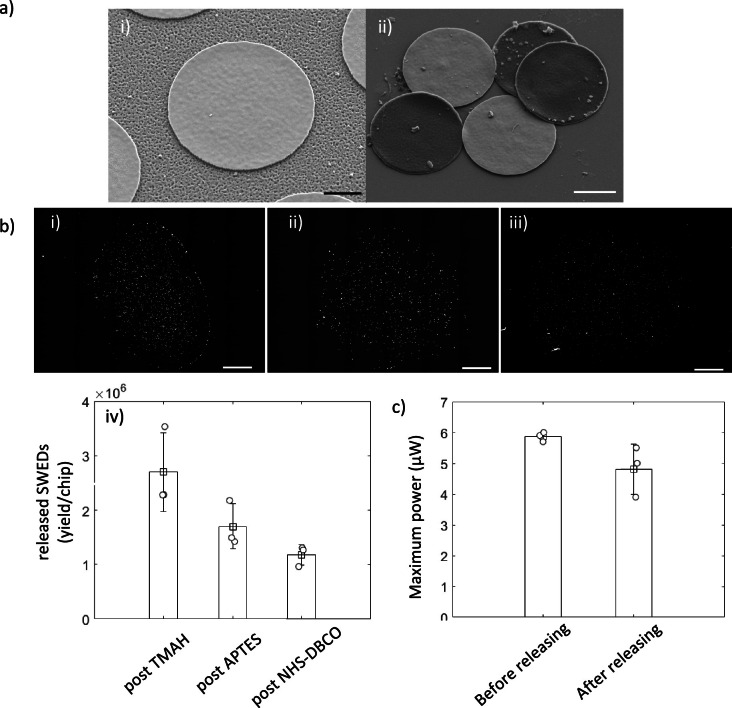
Device release, functionalization and characterization. (**a**) SEM images of the SWEDs (i) before releasing, and (ii) after releasing from the substrate. Scale bar – (i) 2 µm, (ii) 4 µm, n=5. (**b**) Depicting SWEDs collection efficiency at distinct functionalization stages. Each 4" silicon wafer was segmented into 12 chips, each measuring 20 mm x 20 mm and housing approximately 2.8 million SWEDs with a diameter of 10 µm prepared for release. The figure showcases the yield of released SWEDs following (i) the TMAH release step, (ii) the APTES and (iii) the NHS-DBCO functionalization process. Additionally, (iv) a histogram demonstrates the collection yield, providing a quantitative view of the collection efficiency at each step; values represent mean ± SD (n=3 independent experiments), scale bar − 500 µm. (**c**) Maximum power generated by the devices before and after releasing from the substrate using diluted TMAH solution. Device size – 200 µm in diameter, device structure – PEDOT:PSS|P3HT:PCBM|Ti, intensity – 10 mW/mm^2^ (520 nm); values represent median ± SD (n = 3 devices). Source data

To fit and freely move inside the vasculature without clogging, the size of Circulatronics devices must be similar to or smaller than that of the circulating cells (for reference, a circulating cell such as monocyte has a diameter of 12–18 µm). Hence, we set out to develop subcellular-sized wireless electronic devices (SWEDs) that are free-floating and that can convert extracorporeally applied fields to electrical energy to enable electrical neuromodulation. While there are different modalities for wireless energy harvesting (such as radio frequency, electromagnetic, optical or acoustic^21^) each with its unique characteristics, we decided to use the photovoltaic principle, which involves wireless powering via optical fields. This is because optical modalities provide high spatio-temporal resolution, penetration depth of several centimeters in the human head with intact skull and have already been used for clinical studies (Supplementary Note). Moreover, photovoltaic devices generate d.c. potentials^22^ eliminating the need for any rectifying circuits (saving on-chip area and avoiding circuit complexity). Other modalities can also be employed in future for Circulatronics technology, based on user-defined requirements. Although photovoltaics have been applied previously for neuromodulation^23^, this study investigates subcellular-sized free-floating photovoltaic devices compatible with circulation through bloodstream for in vivo electrical brain stimulation. Moreover, none of the previous photovoltaic devices or implants with other modalities (optical, electrical, radio frequency, magnetic or acoustic) have been demonstrated for brain stimulation with high spatial resolution without surgery. We used organic semiconductors^24,25^ to leverage the photovoltaic effect, as they have unique advantages such as narrow bandwidth for enabling multiplexing, high optical absorption coefficients, mechanical flexibility allowing good interface with soft biological systems and biocompatibility. They also provide ease of fabrication and compatibility with complementary metal-oxide-semiconductor back-end-of-line processing^26,27^ creating opportunities for integrating advanced functionalities in the future (Supplementary Note). The equivalent electrical circuit of these photovoltaic devices consists of a current source (representing optical intensity dependent polaron generation), three diodes and several resistors (Extended Data Fig. 1a). Our devices consist of a three-layer structure: anode, binary blend of semiconducting organic polymers (acceptor and donor material forming the active layer where the excitons are generated) and cathode (Fig. 2a). By customizing the organic polymeric materials, these devices can be tuned to different optical wavelengths that will allow their independent control, enabling multiplexing. To achieve this, we used two different donor materials, poly(3-hexylthiophene) (P3HT) and poly(2,6-(4,4-*bis*-(2-ethylhexyl)-4*H*-cyclopenta(2,1-b;3,4-b′)dithiophene)-alt-4,7(2,1,3-benzothiadiazole)) (PCPDTBT), as their absorption spectra are complimentary to each other (Extended Data Fig. 1b). (6,6)-phenyl-C61-butyric acid methyl ester (PCBM) was used as the acceptor polymer in both cases. Further, poly(3,4-ethylenedioxythiophene):polystyrene sulfonate (PEDOT:PSS) and titanium (Ti) are used as the anode and cathode, respectively, and are chosen based on their work function (Extended Data Fig. 1c) and biocompatibility. To investigate the scaling behavior and gauge the feasibility of subcellular-sized devices, we developed a fabrication process (Methods section ‘Device fabrication’) to create devices with nanoscale thickness (about 200 nm) and different lateral length scales, ranging from diameters of 200 µm (>10 times the diameter of a monocyte) to 5 µm (subcellular size). The nanoscale thickness is critical not only to increase the mechanical flexibility for improved biological interfacing, but also for achieving optimal device performance due to the competing effects of increase in exciton generation as well as recombination effects with the increase in device active layer thickness^28^. These devices can be mass produced at the wafer scale (Supplementary Fig. 1). Figure 2b shows the scanning electron microscopy (SEM) images of the fabricated devices on a 4-inch silicon wafer. The inset shows the zoomed-in image of a single device. The setup for characterizing our devices (Methods section ‘Device characterization setup’) is shown in Fig. 2c. The scaling performance of the devices is illustrated in Fig. 2d. It is seen that these devices, even when scaled down to diameters ≤10 µm achieving subcellular sizes (and less than 0.01× volume of a cell with 12 µm diameter) can generate nanowatts of power. Figure 2e shows the current–voltage characteristics of a SWED (10 µm in diameter) illustrating the increase in short-circuit current (*I*_SC_) generation with the incident optical intensity. There is no major change in the open-circuit voltage (*V*_OC_) even when operating the devices at low intensities, which corroborates the ability of photovoltaics to generate near-constant potential^22^ in the open-circuit condition. Figure 2d,e correspond to P3HT-based devices and the characteristics of the PCPDTBT-based devices are shown in Extended Data Fig. 2. Even at this ultra-small dimension (subcellular sizes), SWEDs generate open-circuit voltages, *V*_OC_ = 0.2 ± 0.008 V (P3HT), *V*_OC_ = 0.17 ± 0.01 V (PCPDTBT) and short-circuit currents, *I*_SC_ = 12.8 ± 2.15 nA (P3HT), *I*_SC_ = 18.2 ± 2.56 nA (PCPDTBT) at incident optical intensity of 10 mW mm^−^^2^. Next, to achieve free-floating SWEDs required for Circulatronics, we developed a process flow to release the structures from the fabrication substrate (silicon wafer) through tetramethylammonium hydroxide (TMAH)-based etching of sacrificial aluminum layer and retrieve and collect them (Methods section ‘Device releasing and collection’ and Extended Data Fig. 3a,b). Our characterizations confirmed that even after the substrate-release process, the devices retain good performance (Extended Data Fig. 3c).

To gain insights on the SWEDs operation in their free-floating form in the extracellular environment, we carried out simulation program with integrated circuit emphasis (SPICE) simulations (Methods section ‘SPICE simulations’). From our SPICE simulations, we estimated that the SWEDs operating point stabilizes at approximately 400 pA and 147 mV (Supplementary Fig. 2). To further characterize the energy harvested by the SWEDs when implanted in the brain, we employed our ex vivo measurement setup allowing probing of the devices and measurement of the generated power, with light penetrating through different brain tissue thicknesses (Fig. 2c). The current–voltage characteristics are plotted in Fig. 2f for various tissue thicknesses at an incident intensity of 24.6 mW mm^−2^ of near-infrared (NIR) light, showing the successful remote operation of the SWEDs (10 µm in diameter) even for the entire mouse brain with the intact skull. The power generated by the SWEDs is plotted as a function of optical intensity for the whole brain without and with the skull in Fig. 2g and as expected the power increases with increase in the incident optical intensity. It is seen that these SWEDs can generate 0.545 ± 0.058 nW and 0.482 ± 0.019 nW of power (for the whole brain without and with the skull, respectively) at an incident optical intensity of 46.06 mW mm^−^^2^ (note that continuous laser illumination up to 100 mW mm^−2^ is demonstrated as safe in vivo, while higher intensities can be used when using pulsed illumination^29^). The ability to independently control the SWEDs using different optical wavelengths when operated through brain tissue is demonstrated in Fig. 2h.

### Autonomous implantation in inflamed brain regions

**Fig. 3:**
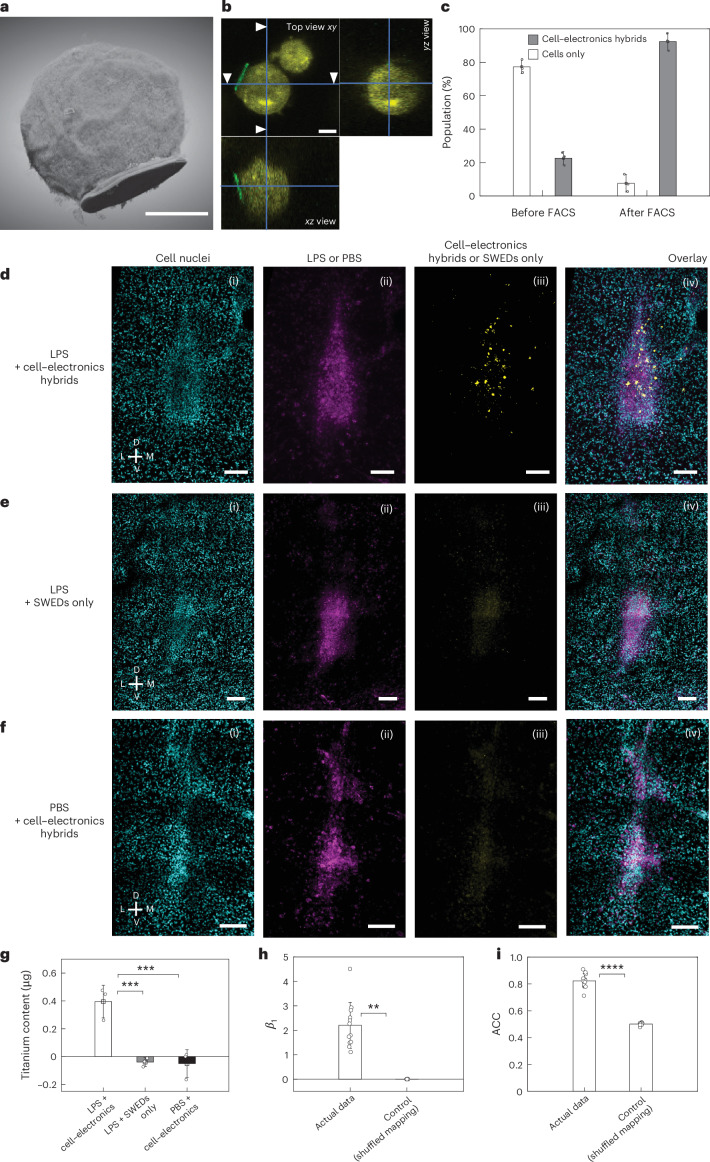
Autonomous implantation of wireless bioelectronics in the brain. **a**,**b**, 3D reconstructed focused ion beam-SEM image (**a**) and confocal z-stack image (**b**) (with cross-sections from various viewing planes) showing a SWED (10 µm in diameter) attached to the immune cell (monocyte, wehi-265.1). Depth resolutions, 20 nm (**a**) and 500 nm (**b**). Scale bars, 5 µm. **c**, Histogram showing the population of cell–electronics hybrids and only cells in the suspension prepared for i.v. injections before and after FACS. A double gating strategy was used to isolate the cell–electronics hybrids from the suspension with a purity of 92.4% ± 5.2%. Values represent mean ± s.d. (*n* = 3 independent experiments). **d**, Representative confocal images of mouse brain slice for the experimental group (*n* > 5 mice) in which cell–electronics hybrids were i.v. introduced in the mice with the target (inflamed) brain region (ventrolateral thalamic nucleus (VL), induced by LPS). Image illustrating (i) Hoechst staining, (ii) target region, (iii) self-implanted cell–electronics hybrids and (iv) overlay image showing localized cell–electronics hybrids self-implanted at the target region. **e**, Representative confocal images of mouse brain slice for the control group (*n* > 5 mice) with same conditions as **d** except that cell–electronics hybrids were replaced by SWEDs alone (without the cells). Image illustrating (i) Hoechst staining, (ii) target region, (iii) SWED channel and (iv) overlay image showing no discernible self-implanted SWEDs. **f**, Representative confocal images of mouse brain slice for the control group (*n* > 5 mice) with same conditions as **d** except that LPS was replaced by PBS. Image showing (i) Hoechst staining, (ii) injected PBS, (iii) cell–electronics hybrids channel and (iv) overlay image showing no discernible self-implanted cell–electronics hybrids. Scale bar, 100 µm for (i)–(iv) in **d**–**f**. **g**, Titanium content in the brain for quantification of the self-implanted SWEDs that contain a titanium layer (after subtracting the intrinsic baseline titanium content in mouse brain) measured by inductively coupled plasma-mass spectrometry when 2 × 10^6^ number of SWEDs only or cell–electronics hybrids were injected into the animal i.v. for the experimental and control groups described in **d**–**f**. A significant increase in titanium content compared to the baseline was observed only for the experimental group while for both the control groups, the changes compared to baseline were not significant, values represent mean ± s.d. (*n* = 3 mice), ***P < 0.001 (one-way analysis of variance (ANOVA) test). **h**, The coefficient (*β*_1_) from the logistic regression model (*n* = 12) suggesting an influence of target region on cell–electronics hybrids localization in the actual experimental data (*β*_1_ = 2.2 ± 0.94), compared to the shuffled control (*β*_1_ = 2.7 × 10^−4^ ± 2.9 × 10^−4^). Values represent mean ± s.d., **P < 0.01 (unpaired two-tailed *t*-test). **i**, The ACC values indicate a better model fit for the experiment (*n* = 12) (ACC = 0.82 ± 0.05) compared to the shuffled control (ACC = 0.50 ± 0.01), supporting the hypothesis of cell–electronics hybrids localization at the target region. Values represent mean ± s.d., ****P < 0.0001 (unpaired two-tailed *t*-test). D, dorsal; L, lateral; M, medial; V, ventral. Source data

**Extended Data Fig. 4:**
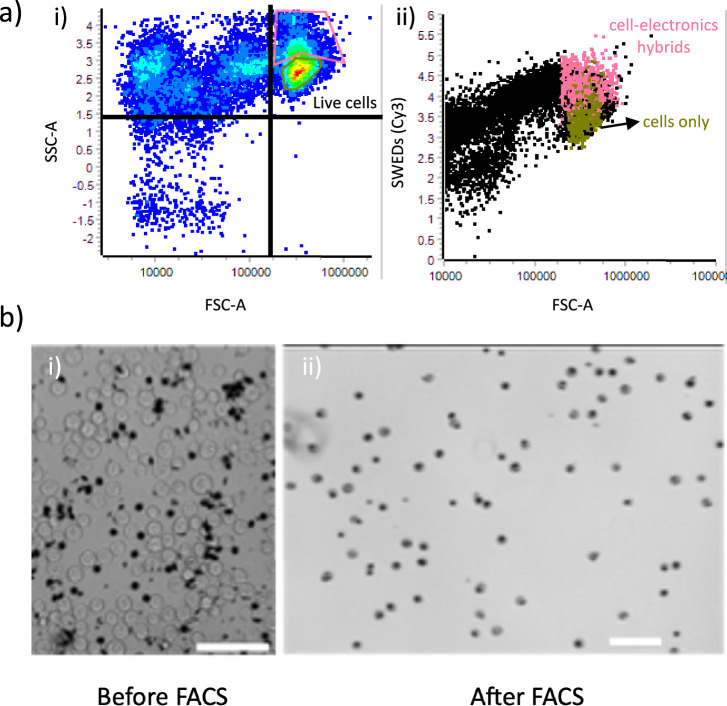
Sorting cell-electronics hybrids using fluorescence-activated cell sorting (FACS). (**a**) Representative flow cytometry gating applied to isolate cell-electronics hybrids from the single-cell suspension (n=3). Live cells are shown in the first quadrant. Legends – SSC-A (side scatter area), FSC-A (forward scatter area), SWEDs were stained with NHS-Cy3. (**b**) Representative images of the cells incubated with SWEDs (i) before and (ii) after cell sorting using FACS. The purity of the sort (cell-electronics hybrids) was found to be 92.4% ± 5.2% (n = 3). Scale bars – 100 µm.

We then investigated whether the SWEDs can be transported by cells through the circulatory system and autonomously implant in the brain. We attached SWEDs onto the surface of immune cells (Fig. 3a,b), specifically monocytes, as they target the region of inflammation^30^ (Methods section ‘Creation of cell–electronics hybrids’) and can cross the blood–brain barrier^31^. We functionalized the surface of monocytes with azide groups, leveraging the available amines on the cell membrane proteins. On the other hand, we functionalized surface of the PEDOT:PSS layer on our SWEDs with dibenzocyclooctyne (DBCO) groups, to attach them to the cells using Click chemistry^32^. Fluorescence-activated cell sorting (FACS) was performed to isolate cell–electronics hybrids with a purity of 92.4% ± 5.2% (Methods section ‘Creation of cell–electronics hybrids’, Fig. 3c and Extended Data Fig. 4). The three-dimensionally (3D) reconstructed focused ion beam-SEM image of the hybrid of a SWED with a monocyte is shown in Fig. 3a while Fig. 3b shows the confocal *z*-stack projection views of the hybrid.

To assess the stability and the transmigration capabilities of the hybrids, we performed transmigration assays (Methods section ‘Transmigration assay’) to characterize the rate of transmigration of various populations (cells, SWEDs and hybrids) and stability of hybrids during the process. The population distribution of the sample for the transmigration can be written as:

$$\begin{array}{l}{{{N}}}_{{\rm{PASS}}}({{t}})=\left((1+{{D}})\times\,{{{n}}}_{{\rm{CELL}}}({{{t}}}_{0})\times\,{{{r}}}_{{\rm{CELL}}}+(1-{{D}})\right.\\\qquad\quad\left.\times\,{{{n}}}_{{\rm{HYBRID}}}({{{t}}}_{0})\times\,{{{r}}}_{{\rm{HYBRID}}}+(1+{\rm{D}})\times\,{{{n}}}_{{\rm{SWED}}}({{{t}}}_{0})\times\,{{{r}}}_{{\rm{SWED}}}\right)\times\,{{t}}\end{array}$$

where *N*_PASS_(*t*) is total transmigration population at a given time *t*, *D* is the fraction of the hybrids that dissociate during the transmigration process, *r*_CELL_ is the rate of transmigration of monocytes (14.14 ± 6.4 mm^−2^ h^−1^, measured experimentally), *r*_HYBRID_ is the rate of transmigration of hybrids, *r*_SWED_ is the rate of transmigration of SWEDs (0.44 ± 0.27 mm^−2^ h^−1^, measured experimentally), *n*_CELL_(*t*_0_) is the number of monocytes, *n*_HYBRID_(*t*_0_) is the number of hybrids and *n*_SWED_(*t*_0_) is the number of SWEDs at *t* = 0 (before transmigration).

**Extended Data Fig. 5:**
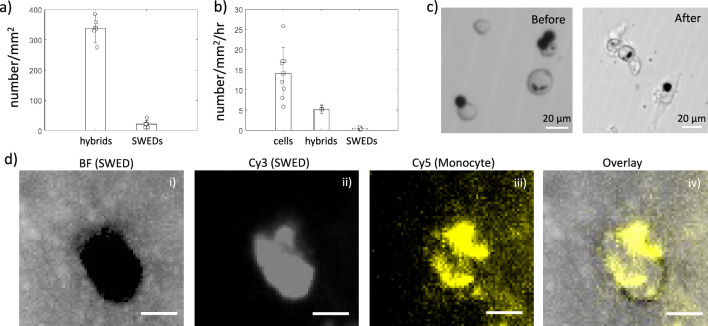
Transmigration Assay, and hybrids stability. (**a**) Histogram showing the number of cell-electronics hybrids and SWEDs crossing the primary endothelial monolayer cultured on top of a porous membrane during the diapedesis process. It can be observed that the hybrids have higher efficacy to transmigrate compared to SWEDs alone corroborating to the importance of creating cell-electronics hybrids. Monocyte Chemoattractant Protein-1 (MCP-1) at a concentration of 200 ng/mL was used for the transmigration process. Values represent mean ± SD; n=4 independent experiments for hyrbids, n=5 independent experiments for SWEDs. (**b**) Histogram showing the differential rate of transmigration for cells, cell-electronic hybrids and SWEDs. Values represent mean ± SD; n=8 independent experiments for cells, n=5 independent experiments for SWEDs. A total of 0.25 million cells/hybrids/SWEDs were plated for the transmigration studies for (**a**) and (**b**). (**c**) Cell-electronics hybrids imaged before and after diapedesis process during the transmigration assay in-vitro, n=4 and (**d**) Zoomed-in representative image showing the (i) SWED (brightfield image), (ii) SWED (fluorescent image), (iii) pre-stained cell and (iv) the overlay image of the self-implanted cell-electronics hybrids at the inflammation (target) site in brain, n=3. Note: The cell-electronics hybrids were formed prior to monocyte staining, leading to non-uniform staining of the monocyte as can be seen in (iii) due to pre-occupation of the cell membrane at the site attached to the SWED. Scale bar – 4 µm. Source data

By counting the proportion of various populations before (at time 0) and posttransmigration (at time *t*), and equating the coefficients using the above equation, we found that a substantial population (86.9% ± 0.9%) of the hybrids remained stable and did not dissociate during the various stages of the transmigration process (Extended Data Fig. 5a–c) and transmigrated at an average rate of 5.24 ± 0.98 mm^−2^ h^−1^.

**Extended Data Fig. 6:**
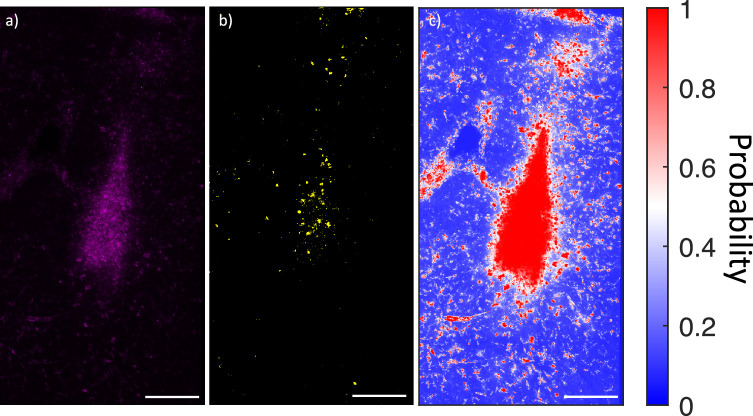
Experimental and predicted images of self-implanted cellelectronics hybrids colocalization with target region. (**a**) Experimental data showing the target brain region (FITC-conjugated LPS). (**b**) Experimental data showing the distribution of self-implanted cell-electronics hybrids. (**c**) Predicted image generated using the logistic regression model, showing the expected cell-electronics hybrids distribution based solely on the intensity values extracted from image (**a**). The color bar ranges from 0 to 1, representing the predicted probability of cell-electronics hybrids in the image. Scale bar – 200 µm for (**a**)-(**c**). The images provide a visual comparison of experimental versus predicted cell-electronics hybrids localization, highlighting the influence of target region on self-implantation of cell-electronics hybrids as suggested by the model.

To investigate whether these hybrids can be self-implanted in the inflamed brain region, we used a classical inflammation model in a deep brain region (ventrolateral thalamic nucleus) of mouse (Balb/C, Methods section ‘Subjects used for animal experiments’) through stereotactic injection of lipo-polysaccharide (LPS) (Methods section ‘Stereotactic injection for the inflammation model’). The dye allowed us to precisely locate the region of LPS injection later during postmortem imaging. The stereotactic surgery is done to emulate the inflammation, and application of our technology does not require any surgery. Next we administered the high purity suspension of cell–electronics hybrids (Fig. 3c and Extended Data Fig. 4b) through i.v. injection (Methods section ‘i.v. injection’). Then 72 h after the i.v. injection, the mice were transcardially perfused, their brains were harvested, fixed and brain slices were prepared for imaging (Methods section ‘Perfusion and imaging’). Our data show that the hybrids self-implanted in the brain (Fig. 3d,g and Extended Data Fig. 5d). In one control group, where only SWEDs were i.v. injected without attachment to immune cells with all other conditions remaining the same as the experimental animals, SWEDs were not observed in the brain images (Fig. 3e), demonstrating the role of immune cells for effective self-implantation. Another control group was studied under the same conditions as the experimental animals except that LPS was replaced with phosphate-buffered saline (PBS), and no hybrids were detected in the brain images (Fig. 3f) corroborating that the cell–electronics hybrids selectively enter the inflamed brain region. Quantification of the number of SWEDs (10 µm) implanted in the brain (Fig. 3g and Methods section ‘Quantifying cell–electronics hybrids in tissue’) found ~14,029 ± 4,154 (*n* = 3 mice) SWEDs installed in the brain of experimental animals. In the two control groups, the number was negligible (Fig. 3g). Further, to evaluate the colocalization of self-implanted cell–electronics hybrids in the region, we used logistic regression analysis (Methods section ‘Assessment of cell–electronics hybrid localization’), which showed that the target region is a determining factor in predicting the self-implantation of cell–electronics hybrids (Extended Data Fig. 6). The high coefficient (*β*_1_, actual 2.2 ± 0.94; *β*_1_, shuffled 2.7 × 10^−4^ ± 2.9 × 10^−4^; *P* < 0.01) (Fig. 3h) and a high accuracy (ACC) value (ACCactual = 0.82 ± 0.05; ACCshuffled = 0.50 ± 0.01; *P* = 0.000009) (Fig. 3i) of the actual experimental data compared to the shuffled control further confirmed the prediction.

### Brain region-specific stimulation

**Extended Data Fig. 7:**
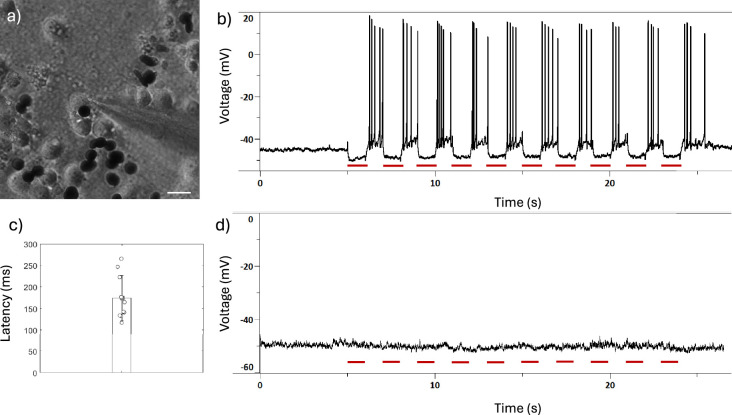
In-vitro patch clamp. (**a**) Optical image showing the patched neuron with a SWED. Scale bar – 20 µm, n=5. (**b**) Representative plot showing current clamp recordings from a neuron during stimulation using SWEDs. The black trace represents the membrane potential recorded in current clamp mode, displaying robust action potential firing in response to optical pulses. Each optical stimulus reliably triggers a burst of action potentials, demonstrating consistent SWED-induced neuronal activation. (**c**) Latency of induced action potentials to the optical pulse offset. Values represent mean ± SD (n=10 measurements); and (**d**) Representative control data showing the membrane potential of a neuron when only optical pulses were applied in the absence of SWEDs. The black trace represents the membrane potential recorded in current clamp mode. Light intensity – 30 mW/mm^2^, n=5 neurons for (**b**) and (**d**). Red dashed line – optical pulse for (**b**) and (**d**). Source data

Next we explored the capabilities of SWEDs to stimulate specific regions of brain. First, to characterize the neuromodulation capabilities of SWEDs, we conducted in vitro patch-clamp experiments by drop-casting SWEDs in neuronal cultures and applied optical illumination (Extended Data Fig. 7a and Methods section ‘In vitro patch-clamp experiments’). These studies revealed reliable light-triggered neuromodulation capabilities of our SWEDs. Our patch-clamp recordings demonstrate that a ten-pulse optical train consistently induces robust neuronal firing patterns, with each stimulation pulse reliably triggering multiple action potentials. The generated action potentials show precise temporal correlation to optical pulse offset (Extended Data Fig. 7b,c). Our control experiments (Extended Data Fig. 7d) confirm that optical illumination alone (without SWEDs) showed minimal membrane perturbations and does not elicit neuronal responses, validating that the observed effects are specifically due to SWED activation rather than nonspecific heating due to light or other optical artifacts.

**Fig. 4:**
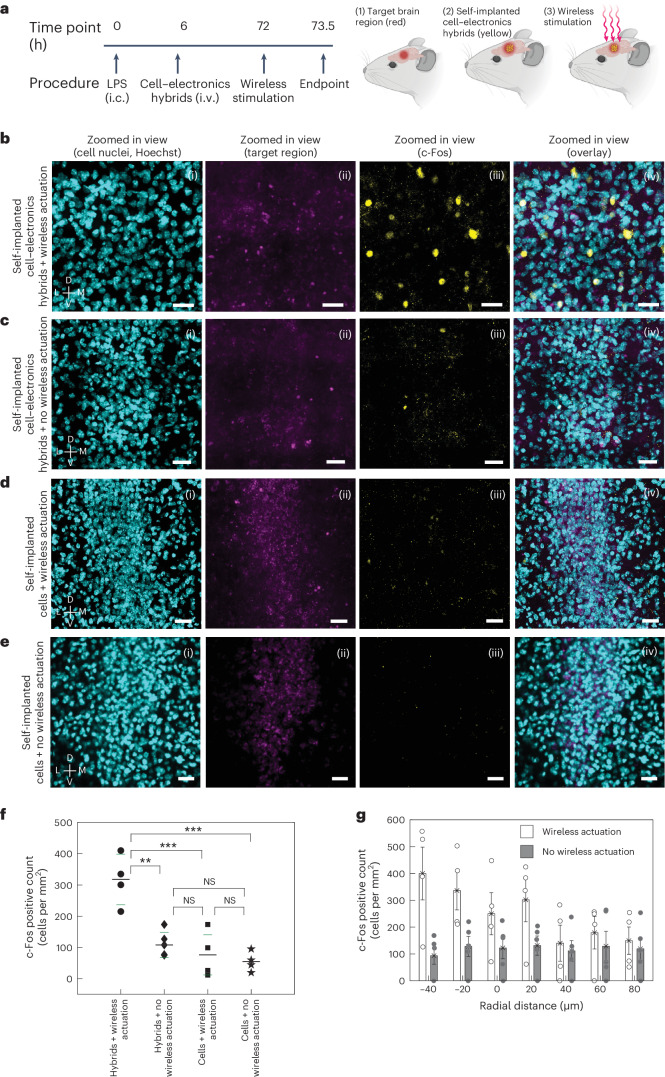

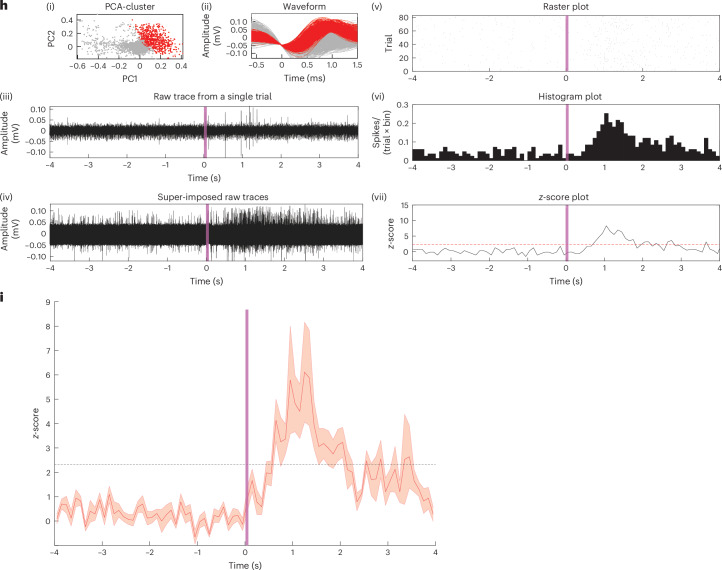
Nonsurgical targeted focal brain stimulation. **a**–**e**, Schematic diagram showing the timeline and the wireless stimulation scheme (**a**). Representative images showing the (i) cell nuclei, (ii) target region, (iii) c-Fos activity and (iv) overlay confocal images for mice with self-implanted cell–electronics hybrids for the experimental group where optical actuation was applied (*n* = 4 mice) (**b**); and control groups where self-implanted cell–electronics hybrids were present but no optical actuation was applied (*n* = 5 mice) (**c**), where only cells were self-implanted and optical actuation was applied (*n* = 5 mice) (**d**) and where only cells were self-implanted and no optical actuation was applied (*n* = 4 mice) (**e**). The optical pulse sequence applied in **b** and **d**: 792 nm, 15 mW mm^−^^2^, 10-ms pulse width, 20 Hz, 20-min duration. Scale bars, 25 µm for (i)–(iv) for **b**–**e**; SWED structure used for **b** and **c** PEDOT:PSS|PCPDTBT:PCBM|Ti|TiN. **f**, Scatter plot showing the number of c-Fos positive cells in the target region for the experimental (self-implanted hybrids with wireless optical actuation) and control groups (self-implanted hybrids without optical actuation, self-implanted cells with wireless optical actuation and self-implanted cells without wireless optical actuation). Longer (shorter) horizontal lines represent the median (standard deviation) of the data; *n* = 4 mice (hybrids + wireless actuation, and cells + no wireless actuation), *n* = 5 mice (hybrids + no wireless actuation, cells + wireless actuation), **P < 0.01, ***P < 0.001; one-way ANOVA test; NS, not significant. **g**, Bar plot showing the distribution of c-Fos positive cells as a function of radial distance from the target region boundary (represented by 0 on the *x* axis; positive and negative values on the *x* axis correspond to regions outside and inside the target, respectively) for the experimental (cell–electronics hybrids + wireless actuation) and control (cell–electronics hybrids + no wireless actuation) groups, *n* = 4 mice (experimental group), *n* = 5 mice (control group), values represent mean ± standard error of the mean (s.e.m.). **h**, (i) Principal component analysis (PCA) cluster, (ii) spike waveform, (iii) representative raw trace from a single trial, (iv) super-imposed raw-traces from multiple trials, (v) raster-plot displaying spike timing across multiple trials, (vi) peri-stimulus time histogram and (vii) *z*-score of the representative recorded unit for the experiment when hybrids were self-implanted and NIR light was applied. The vertical shaded line ((iii)–(vii)) shows time at 0–100 ms for the optical pulse. Horizontal dashed line (in *z*-score plot): *z* = 2.33; bin, 100 ms, Optical pulse: 100 ms, 15 mW mm^−^^2^, 792 nm. **i**, Normalized ensemble activities (*z*-scores) of active units in the experimental dataset (LPS + hybrids + NIR light) (*n* = 14, 5 mice). Data are presented as mean ± s.e.m., vertical shaded line shows time at 0–100 ms for the optical pulse. i.c., intracranial. Illustrations in **a** created using BioRender.com. Source data

Building on these in vitro findings, we next characterized brain stimulation in vivo using c-Fos immunohistochemistry (IHC), which is widely used to delineate cell activity^33,34^ and can provide information on spatial distribution of the stimulated region. For this, 18 mice were divided into experimental and control groups to assess SWED-mediated neuromodulation specificity (Fig. 4a). Nine mice received i.v. injections of cell–electronics hybrids (SWED structure PEDOT:PSS|PCPDTBT:PCBM|Ti|TiN, 10-μm diameter) for brain self-implantation, while nine received monocytes alone. After 72 h, mice were organized into 4 groups: hybrids with wireless actuation (*n* = 4), hybrids without actuation (*n* = 5), monocytes with actuation (*n* = 5) and monocytes without actuation (*n* = 4). NIR light actuation (792 nm, 15 mW mm^−2^, 10 ms pulses, 20 Hz) was applied for 20 minutes. Figure 4b–e shows representative confocal images of c-Fos activation in the brain region of experimental and control mice. Quantification of the brain tissue, using a pipeline optimized for counting c-Fos positive cells in tissues (Methods section ‘In-vivo c-Fos modulation and analysis’), revealed a statistically significant number of c-Fos positive cells (317.8 ± 80.96 cells per mm^2^, *n* = 4 mice) in the brain region of the experimental mice (hybrids + wireless actuation) compared to the control groups: hybrids + no wireless actuation (107.9 ± 40.57 cells per mm^2^, *n* = 5 mice), cells (monocytes) + wireless actuation (76.2 ± 64.34 cells per mm^2^, *n* = 5 mice) and cells (monocytes) + no wireless actuation (73.38 ± 44.14 cells per mm^2^, *n* = 4 mice) (Fig. 4f). These results collectively demonstrate that the activation of self-implanted cell–electronics hybrids by NIR light specifically induces neural activity, as evidenced by increased c-Fos expression. On the other hand, none of the presence of the monocytes only, the hybrids without NIR light or the NIR light alone is sufficient to elicit these neural changes. In addition, the spatial distribution of c-Fos positive cells showed a distinct pattern of clustering around the target area in mice where hybrids were self-implanted and had received wireless stimulation. Radial distribution analysis revealed a decrease in the density of c-Fos positive cells as the distance from the target locus increased and the c-Fos positive cell density returned to the baseline within a few tens of micrometers outside the target region boundary. This was in contrast with the control animals (which did not receive wireless stimulation), where the activity was uniformly dispersed, as shown in Fig. 4g. Note that in our experiments, light is not required to be localized at the target and is illuminated over a wider region.

**Extended Data Fig. 8:**
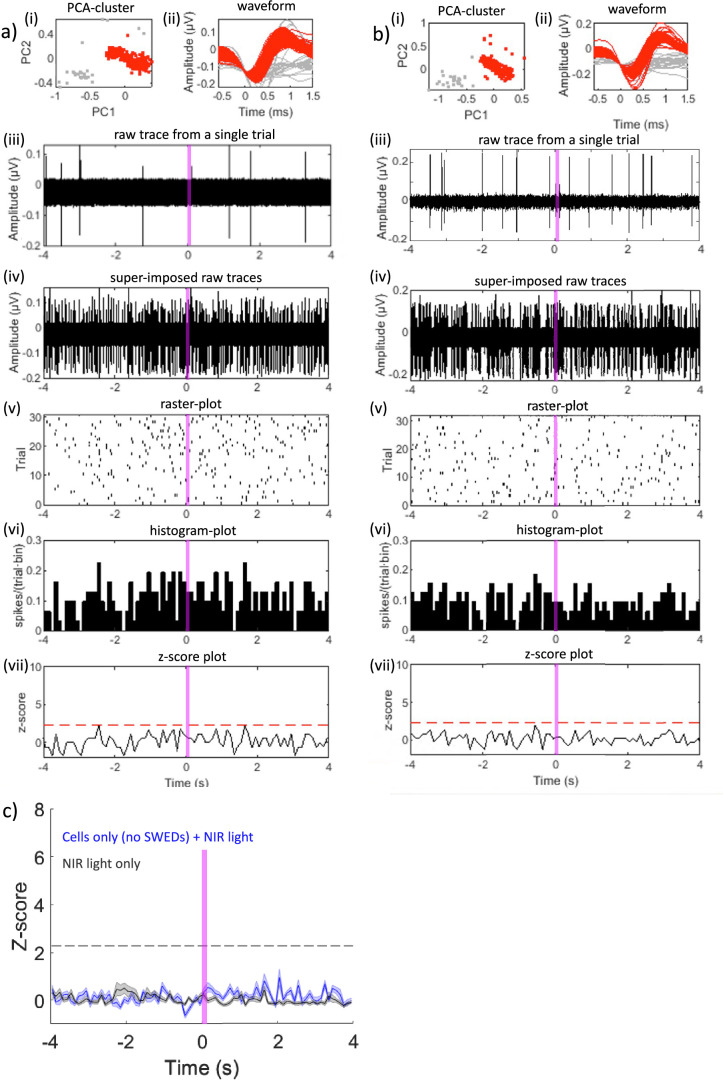
(a) NIR light and (b) LPS + cells + NIR light controls. (i) Principal component analysis (PCA) cluster, (ii) spike waveform, (iii) representative raw trace from a single trial, (iv) super-imposed raw-traces from multiple trials, (v) raster-plot displaying spike timing across multiple trials, (vi) peri-stimulus time histogram and (vii) z-score of the representative recorded unit for (**a**) NIR light and (**b**) LPS + cells + NIR light controls. Vertical shaded line - time = 0–100ms for the optical pulse. Horizontal dashed line (in z-score plot) – z=2.33, bin = 100ms, Optical pulse – 100 ms, 15 mW/mm^2^, 792 nm. (**c**) Normalized ensemble activities (z-scores) of the control datasets (NIR light (n=58, 5 mice) and LPS+Cells+NIR light (n=61, 5 mice)). Power analysis for sample size: β>95%, α<0.01. Data are presented as mean ± SEM, horizontal dashed line – z=2.33, vertical shaded line - time = 0–100 ms for the optical pulse, not significant (n.s.) (student t-test).

**Extended Data Fig. 9:**
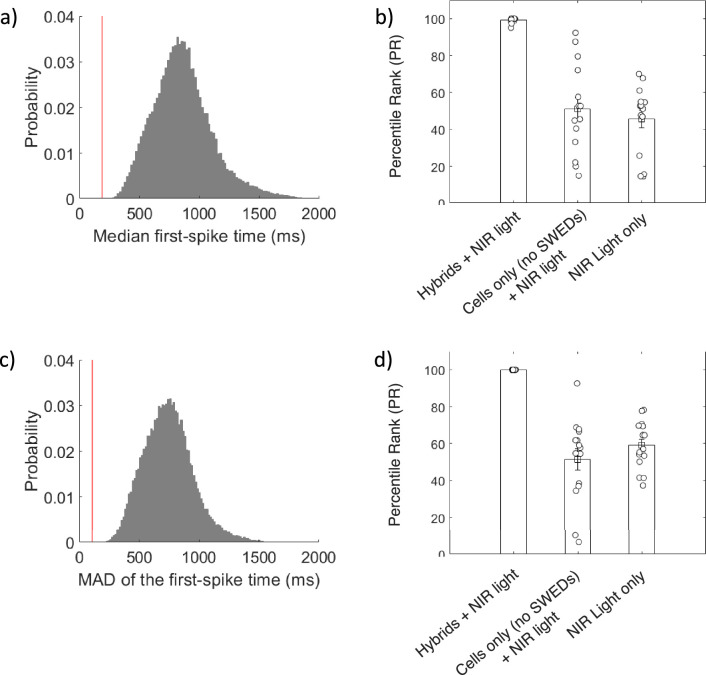
Statistical analysis of spike timing in response to optical pulses. (**a**) Distribution of median first-spike-timing from optical pulse offset obtained from the generated control datasets. The vertical red line indicates the observed median first-spike-timing from optical pulse offset in the representative experimental data, which occurs considerably earlier. (**b**) Bar plot showing the percentile rank (PR) of the actual median spike time within the shuffled distribution for the pooled experimental (Hybrids + NIR light) and control (Cells only (no SWEDs) + NIR light; and NIR Light only) groups. High PR (99.18±0.43) only in the experimental group indicates statistically significant early timing. (**c**) Distribution of median absolute deviations (MAD) of first-spike-timing from optical pulse offset from the 10,000 control datasets (generated using randomization-based resampling method within the baseline period (−3s to 0s)). The red line shows the observed MAD in the representative experimental data, demonstrating tighter temporal clustering of spikes than expected by chance and (**d**) Bar plot showing the high percentile rank of the actual MAD within the distribution for the pooled experimental (Hybrids + NIR light) and control (Cells only (no SWEDs) + NIR light; and NIR Light only) groups. High PR (99.94±0.04) only in the experimental group indicates statistically significant shorter response timing. Data are presented as mean ± SEM for (**b**) and (**d**). Source data

To further characterize the neuronal stimulation in vivo, we carried out single-unit recordings to electrically measure the modulated neural activity in the presence of cell–electronics hybrids during NIR light stimulation (Methods section ‘Single-unit recording’). The recorded units (*n* = 14 out of 64 units, 5 mice) exhibited a statistically significant increase in neural activity with optical pulse stimulation (Fig. 4h and Supplementary Fig. 3). Comprehensive control studies in the ventrolateral thalamic nucleus region with NIR light alone (*n* = 58 units, 5 mice) and in LPS-inflamed ventrolateral thalamic nucleus with self-implanted monocytes (without SWEDs) plus NIR light (*n* = 61 units, 5 mice) confirmed that neuronal activation was specifically attributable to SWED stimulation rather than confounding factors (Extended Data Fig. 8 and Supplementary Fig. 4). Further, statistical analysis of spike timing (Methods section ‘Statistical analysis of spike timing and time locking’ and Extended Data Fig. 9) revealed that experimental group responses exhibited both temporal alignment to optical pulses and consistency exceeding all control cohorts. Compared to NIR light only (45.58 ± 4.84 percentile rank) and cells only + NIR light (51.24 ± 6.15) controls, experimental first-spike latencies relative to pulse offset ranked at the 99.18 ± 0.43 percentile. Temporal consistency showed even greater distinction, with median absolute deviations (MAD) in experimental responses ranking at the 99.94 ± 0.04 percentile versus 59.02 ± 3.36 (NIR only) and 51.47 ± 5.85 (cells only) controls. These quantitative measures provide statistical evidence that the neuronal responses exhibited temporal consistency associated with the optical pulse offset, with both the speed and deviation of responses being notably different from the spontaneous neural activity. Lastly, the pooled *z*-score ensemble data from the recorded active units (Fig. 4i) further demonstrate rapid, transient excitatory neuronal responses triggered only by the activation of cell–electronics hybrids in the presence of NIR light, highlighting the capability of Circulatronics to wirelessly stimulate neurons.

### Biocompatibility studies

**Fig. 5:**
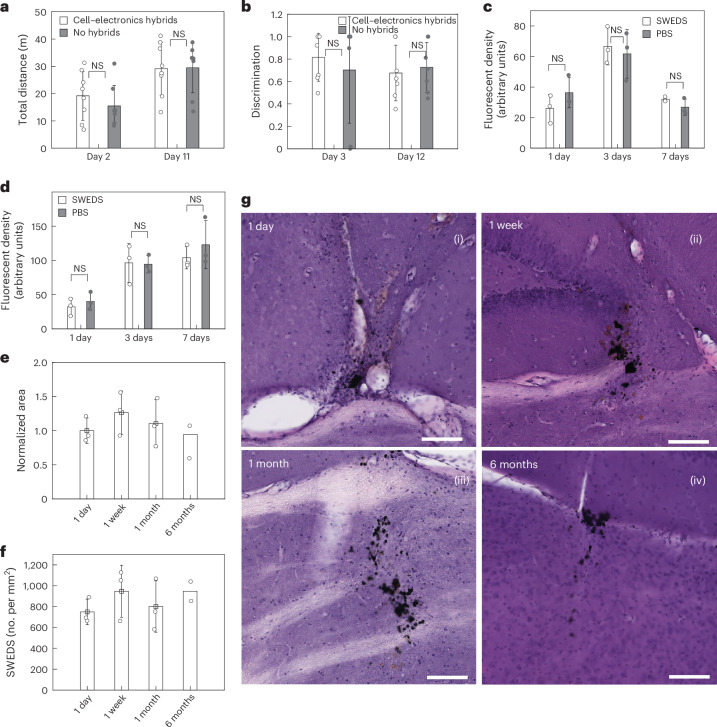
Biocompatibility studies. **a**, Bar plots showing the motor activity of the animal during the OFT with minimal differences between the experimental (with cell–electronics hybrids) and control (without hybrids) animals. Mean ± s.e.m. (*n* = 8 mice, NS, two-way ANOVA test). **b**, Bar plots showing the discrimination scores for the novel object in the NORT with negligible differences between the experimental and control animals. Mean ± s.e.m. (*n* = 8 mice, NS, two-way ANOVA test). **c**,**d**, Iba-1 (**c**) and GFAP levels (**d**) after intracranial injection of SWEDs or PBS in the brain. There was an initial increase in Iba-1 and GFAP densities in the vicinity of the injection site within 3 days after injections. GFAP increase reached a plateau 3–7 days after injections (no statistically significant difference between 3-day and 7-day cases), while a reduction in Iba-1 density after 7 days was apparent. Notably, minimal disparities in astrocyte and/or microglial levels between SWED and PBS injections are discernible, suggesting that the observed immunoreactions of astrocytes and microglia were to the intracranial injection procedure itself and not to the SWEDs. Mean ± s.d. (*n* = 3 mice, NS, two-way ANOVA test for **c** and **d**). **e**,**f**, Bar plots showing the normalized area occupied by SWEDs (**e**) and the density of SWEDs (**f**) up to 6 months postinjection. Mean ± s.e.m. **g**, Representative H&E-stained brain sections for the four time points (1 day, 1 week, 1 month and 6 months postinjection of SWEDs). Scale bars, 100 µm. For **e**–**g**, *n* = 3 mice for each of the 1 day, 1 week and 1 month time points and *n* = 2 mice for the 6-month time point. Source data

**Extended Data Fig. 10:**
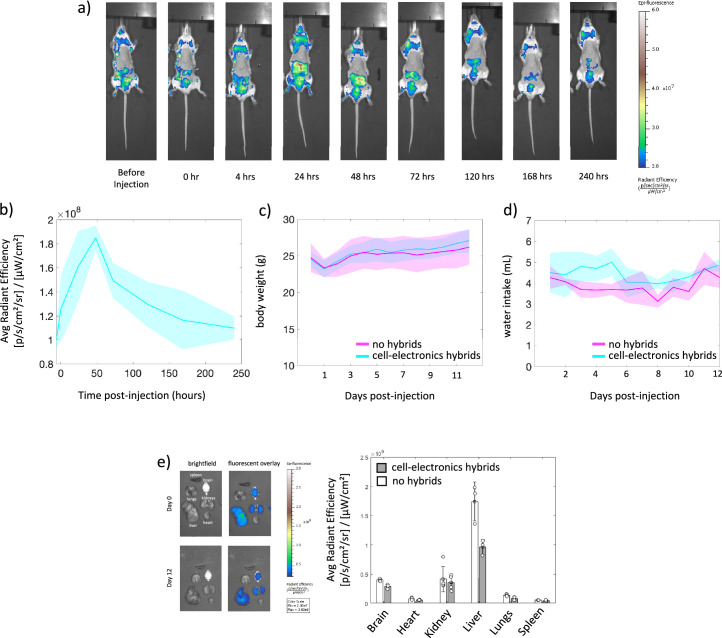
Clearance, continuous health monitoring and biodistribution. (**a**) IVIS images showing the fluorescent cell-electronics hybrids being cleared from the body over a 10-day period (n=3 mice). (**b**) Bar plot showing the radiant efficiency of fluorescently tagged cell-electronics hybrids over a period of time for liver, values represent mean ± SD (n=3 mice). For (**c**)-(**e**) below, experimental group underwent administration of cell-electronics hybrids (2 million) via intravenous injection while controls did not. (**c**) Body weight and (**d**) water intake measured for the animals over the entire experimental duration, values represent mean ± SD (n>3 mice) (**e**) Representative ex-vivo IVIS image and a bar plot showing the radiant efficiency of various organs (Brain, Heart, Kidney, Liver, Lungs and Spleen) at day-12 of the study, values represent mean ± SD (n=3 mice). No significant difference is observed between the experimental and control groups corroborating to the clearance from all these organs (unpaired two-tailed t-test). Source data

Both in vitro and in vivo biocompatibility studies were performed. The biocompatibility of SWEDs was first assessed in vitro against monocytes and cultured primary neurons. The metabolic activity assays (3-(4,5-dimethylthiazol-2-yl)-2,5-diphenyltetrazolium bromide, MTT) for cytotoxicity in monocytes (Methods section ‘Cytotoxicity assay for monocytes’ and Supplementary Fig. 5) and neurons (Methods section ‘Cytotoxicity assay for cultured neurons’ and Supplementary Fig. 6) indicate that the SWEDs do not impair the health or viability of these cells. Following this, the in vivo biocompatibility of our technology was evaluated for both potential short-term and long-term effects (Methods). Animals with the LPS injection in the brain were randomly divided into two groups: the experimental group received an i.v. injection of cell–electronics hybrids, while the control group did not. We performed the complete blood count analysis (Supplementary Table 1) and comprehensive blood serum chemistry analysis (Supplementary Table 2) at 3 days post LPS injection, which revealed no adverse effects from cell–electronics hybrids in the experimental group compared to the controls (Methods section ‘Blood count and blood serum chemistry analysis and histology’). Behavioral tests were conducted to evaluate the impact of cell–electronics hybrids on the animals’ locomotor and cognitive functions by open field test (OFT, at 2-day time point) and novel object recognition test (NORT) (at a 3-day time point), respectively. In OFT, the animals showed minimal differences in the total locomotion distance between the experimental and control group (Methods section ‘OFT’ and Fig. 5a). In NORT, both experimental and control groups showed the same discrimination between novel and familiar objects (Methods section ‘NORT’ and Fig. 5b). Histological assays using hematoxylin and eosin (H&E) staining (Supplementary Fig. 7) for further investigation of systemic effects, showed neither morphological differences nor cytotoxicity in tissues of organs including the heart, liver, lung, spleen and kidney for the experimental group compared to those of the control. Moreover, to understand the potential immune response of the brain to the SWEDs while differentiating from immune response to LPS (which create strong immunoreactions by itself) in the brain tissue, SWEDs were intracranially injected, and the intracranial injection of PBS was used as control. For this study, we conducted IHC on brain tissue, investigating microglia (marker-ionized calcium-binding adapter molecule 1 (Iba-1)) and astrocytes (marker, glial fibrillary acidic protein (GFAP)) (Fig. 5c,d, Supplementary Fig. 8 and Methods section ‘Testing immunoreaction to SWEDs’). Notably, there were no statistically significant differences in the levels of Iba-1/GFAP between SWEDs and PBS injections, corroborating to the biocompatibility of the SWEDs. We also monitored in vivo clearance of the cell–electronics hybrids over time after their i.v. injection using an in vivo imaging system (IVIS) and observed that clearance was achieved (fluorescent signal retuned back to the baseline level) 10 days after injecting cell–electronics hybrids (Extended Data Fig. 10a,b and Methods section ‘Clearance studies of i.v. injected cell–electronics hybrids’). The general health (body count score, water intake and body weight) of the experimental group with the cell–electronics hybrids and the control group without the hybrids was continuously monitored during this period. Both groups maintained a body count score of 3 throughout the study as well as their water intake, and body weight remained within the normal range (Methods section ‘Continuous health monitoring’ and Extended Data Fig. 10c,d). Furthermore, apart from general health monitoring we repeated all the tests mentioned above for these animals after clearance of the cell–electronics hybrids (after day 10). No adverse effects were observed in the experimental group compared to the control group for the complete blood count analysis and comprehensive blood serum chemistry analysis as well as behavioral tests (OFT and NORT) (Supplementary Tables 1 and 2 and Fig. 5a,b). Moreover, no adverse systemic effects were evidenced by histology studies of different organs after euthanizing the animals (Supplementary Fig. 7). Ex vivo IVIS imaging of the different organs also confirmed that clearance was successfully achieved from all major organs (Extended Data Fig. 10e). We found that the SWEDs by themselves, when not attached to the cells (they were directly injected into the brain for this study), did not exhibit any decrease in number or spread in the area of occupation in the brain region (tested up to 6 months) (Fig. 5e,f). Furthermore, we found no cytotoxic effects from the SWEDs from the H&E staining of the brain during this 6-month period (Methods section ‘H&E studies of brain tissues’ and Fig. 5g).

## Discussion

We have demonstrated that a bioelectronic implant for the brain, which is guided by immune cells’ native tropism to inflammation sites, can autonomously implant without external intervention and enables wirelessly controlled, focal brain stimulation. To achieve this technology, we first developed subcellular-sized, free-floating electronic devices that harvest optical energy with record power conversion efficiency of 0.18% ± 0.02%: 4 orders of magnitude higher than any previous device^35^ while remaining small enough to traverse the vasculature (Supplementary Table 3). We then coupled these to immune cells via click chemistry and showed that the hybrids travel through the vasculature, self-implant at sites of inflammation and can be wirelessly actuated for focal stimulation at deep brain regions such as the ventrolateral thalamic nucleus in the mouse brain. Based on the target disease of interest, appropriate cells can be selected. We have chosen the inflamed region in the brain as our target, as targeted electrical modulation of the region of inflammation can address the inherent mechanisms underlying a broad spectrum of diseases ranging from neurodegenerative diseases to stroke, cancer, neuropathic pain and nerve injury^11–20^. While optical modality for wireless energy harvesting is used here, it would be interesting to explore other modalities in the future based on application needs, employing the generalized principle of Circulatronics and cell–electronics hybrids demonstrated here. Future work can also involve increasing the self-implantation efficiency of the devices through processes such as surface functionalization, cell engineering, cell activation and optimizing injection routes^36,37^. In addition, future work can involve tuning the devices for chronic or transient use, based on the specific applications. This can be achieved through the integration of different material technologies into the devices to tune their longevity as needed and allow their degradation into physiologically and environmentally benign by-products after the required application period is over, to avoid the need for explantation or extraction of the devices. For applications where SWEDs will be required for longer time in the brain target region, they can be designed in future to be released from cells after reaching the target, using attachment linkers that either self-degrade after a few days or are cleavable with external fields (such as widely available light cleavable linkers) or specific biological cues (such as pH, proteases, other biomolecules)^38,39^. Further, Circulatronics can be applied to different diseases by adapting to the specific electrical stimulation protocols via modulating the external light source and not requiring the addition of complex circuitry to the SWEDs. While current work focuses on stimulation, future endeavors could enable sensing and data analysis capabilities. As SWED is complementary metal-oxide-semiconductor back-end-of-line compatible, future implementations can employ improved sensors and nano-transistors (Supplementary Note). All the required power can be provided by the onboard SWED power source that can wirelessly harvest energy from transmitters present outside the body (Supplementary Note). This could lead to future technologies for brain stimulation and recording with data analysis and feedback capabilities on-chip (in the wireless self-implant itself), harnessing the capabilities of nanoelectronics without any surgery.

## Methods

### Device fabrication

The devices were fabricated on two substrates: 4" silicon wafer (p-doped, <100>, single-side polished) and indium-tin-oxide (ITO) coated glass substrate. Silicon wafers were used as bought from University Wafers and a sacrificial layer of titanium and aluminum (100 nm and 200 nm) was deposited using electron-beam evaporation. Thereafter, the wafers were plasma treated (oxygen, 150 W, 60 s) to improve the wettability for spin-coating process. The ITO-coated glass substrates were bought from Sigma, and cleaned by sonicating in deionized (DI) water, acetone and iso-propyl alcohol, respectively, for 10 min each and plasma treated (oxygen, 150 W, 60 s) before spin-coating. PEDOT:PSS (Ossila) was used as bought. 5% dimethyl sulfoxide (DMSO, Sigma, to increase the conductivity), 0.2% (3-glycidyloxypropyl)trimethoxysilane (Sigma, for higher water stability) and 0.2% Dynol 604 (to improve the wettability of the solution while spin-coating) were added to the PEDOT:PSS solution and stirred for 30 min before use. After mixing, the solution was filtered through a 0.2-µm polyethersulfone syringe filter. Thereafter, this solution was spin-coated (1,000 rpm, 60 s) on the substrates (silicon or ITO-coated glass substrate) and annealed at 120 °C for 10 min. The preparation of the organic semiconducting blends P3HT:PCBM or PCPDTBT:PCBM was performed in a glovebox under nitrogen atmosphere. The binary blends were prepared at least 6 h before spin-coating them on the Si wafers or ITO-coated glass substrate. P3HT:PCBM was prepared in a ratio of 1:0.83 with the overall concentration of 22 mg ml^−1^. Similarly, PCPDTBT:PCBM was prepared in a ratio of 1:2 with the overall concentration of 22 mg ml^−1^. While P3HT:PCBM was spin-coated at 800 rpm (100 ± 12 nm), PCPDTBT:PCBM was spin-coated at 1,200 rpm (80 ± 15 nm) for 60 s (only 1 polymer blend at a time) on top of PEDOT:PSS and annealed at 130 °C for 30 min. Once the spin-coating was done, the samples were transferred to the electron-beam evaporation machine for deposition of the top metal electrode (titanium (Ti): 50 nm, 1 Å s^−1^). For samples requiring titanium nitride (TiN) (for improved electrode–electrolyte interface), it was deposited after Ti deposition using reactive sputtering (30 nm, 0.27 Å s^−1^, argon:nitrogen at a 3:1 ratio). Once Ti/TiN was deposited, wafers were kept under vacuum until the lithography process. For photolithography, the samples were dehydrated at 100 °C for 30 min to increase the adhesion of the photoresist (AZ3312). Photoresist was spin-coated (3,000 rpm, 60 s) on the substrates, prebaked at 100 °C for 60 s and exposed using MLA150 (Heidelberg, dose 130 mJ cm^−2^ at 375 nm). The samples were postbaked at 100 °C for 60 s and then developed using AZ300-MIF developer for 60 s. After developing the photoresist, samples were hard baked on a hotplate for 30 min at 100 °C. For patterning the devices, the substrates were then transferred for dry etching of Ti/TiN and the organic polymers in an Oxford-100 reactive ion etcher. Ti/TiN was etched using SF_6_ plasma (200 W, 3.5 min). After the Ti/TiN etch, the samples were sonicated in acetone for 5 min to remove the photoresist and its residuals. After this, oxygen plasma was used to etch away the organic polymers as well as the left-over photoresist (Ti/TiN served as a hard mask for this step). The wafer was cleaved into small chips (20 mm × 20 mm) and stored under a nitrogen environment for further use.

### Device characterization setup

A custom-built probe station was used for characterizing the devices (200 µm to 5 µm in size). External laser sources (50 mW, 520 nm, Thorlabs; 100 mW, 785 nm, Coherent) were coupled into the probe station for providing a bottom illumination onto the devices from the PEDOT:PSS side. For the ex vivo measurements (792 nm laser, HJ Optronics was used) the light passed through the brain before being incident on the devices. The micromanipulators were connected to a potentiostat (CompactStat, Ivium Technologies) for measuring the current–voltage curves. An upright optical microscope was used to locate the devices on the substrates and aligned with the bottom light source each time before recording the measurements.

### Device releasing and collection

The devices fabricated on the silicon wafers had to be released to create free-floating devices for Circulatronics technology. For this, the wafer was cleaved into smaller chips and put inside a 5-ml glass vial. 1.5 ml of diluted TMAH solution (2.7% v/v) was used for a 20 mm × 20 mm silicon chip. The glass vial was then put in a sonication bath for 10 min to etch away the sacrificial aluminum layer and collect the devices. Thereafter, the devices were rinsed multiple times (using a custom vacuum-based filtration setup) in deionized water to get rid of trace amounts of TMAH. The devices were collected and stored for further experiments (Extended Data Fig. 3).

### SPICE simulations

SPICE simulations were performed to investigate the SWED’s operation in free-floating form in the extracellular environment and to understand the effect of seal resistance (*R*_SEAL_) on the induced transmembrane potentials. The circuit model used for the simulations included several key components. Experimentally measured current–voltage characteristics of a SWED were fitted using third-order polynomials, a linear resistance and a shunt resistance. The electrode–electrolyte interface impedances for the two device terminals were extracted using Electrochemical Impedance Spectroscopy measurements performed with a potentiostat (IviumStat) and incorporated into the circuit model to accurately represent the electrode–electrolyte interface: *R*_TiN_/*C*_TiN_ − 2.14 GΩ/214.3 pF, *R*_PEDOT:PSS_/*C*_PEDOT:PSS_ − 1.6 GΩ/583 pF. The cell membrane was modeled as a parallel *RC* circuit, consisting of the membrane’s resistance and the capacitance. The resistance and capacitance values (*R*_CELL_, *C*_CELL_) for the cell were taken from the literature^23^ to ensure a realistic representation of the neural membrane. *R*_SEAL_ represents the leakage resistive path between the SWED and the cell, which can vary depending on the interface conditions. In the simulations, *R*_SEAL_ was varied from 1 kΩ to 100 MΩ to simulate different interface conditions and to study its effect on the induced transmembrane potentials. The simulations were carried out using the LTspice XVII software (Linear Technology). The operating point of the SWEDs and the induced transmembrane potentials were determined for each *R*_SEAL_ value by analyzing the simulation results. From the simulations, we estimated that the SWEDs’ operation point stabilizes at approximately 400 pA and 147 mV (Supplementary Fig. 2b) in the extracellular environment. Moreover, the simulations shed light on the relationship between *R*_SEAL_ and the induced transmembrane potentials, indicating that high *R*_SEAL_ values—such as 100 MΩ (typical of neural interfaces with multi-electrode arrays)—enable SWEDs to induce transmembrane potentials exceeding the threshold voltage (13.5 ± 2.8 mV, determined experimentally using whole-cell patch clamp, Supplementary Fig. 10 and Methods section ‘Threshold voltage estimation for neurons’) required for neuronal activation (Supplementary Fig. 2c).

### Threshold voltage estimation for neurons

To determine the threshold voltage necessary for action potential initiation in primary embryonic rat hippocampal neurons, we used whole-cell patch-clamp recordings. Neurons cultured in vitro were subjected to constant current injections, and the transmembrane potential (*V*_M_) was measured in current-clamp mode. The threshold for action potential generation was identified by analyzing the d*V*_M_/d*t* for a nonlinear increase indicative of ion channel activation. Specifically, during the initial period of charge injection, we calculated the mean and standard deviation of d*V*_M_/d*t* when it was expected to remain constant. The onset of nonlinearity, marking the neural threshold, was determined when d*V*_M_/d*t* exceeded the mean by 2.5 times the standard deviation, and continuing to rise thereafter. This method yielded an estimated action potential threshold of 13.5 mV ± 2.8 mV across the sampled neurons (*n* = 9), providing a quantitative basis for understanding neuronal excitability under controlled stimulation conditions.

### NIR light transmittance measurements

A continuous-wave diode laser, operating at 792 nm, was used for transmittance evaluations. Brain slices of varied thicknesses were obtained from a perfused mice, while a tissue phantom was synthesized following the protocol used in ref. ^40^. For measurements, the sample (brain slice or brain phantom) was placed between the laser source and a photodetector. Transmittance was quantified by comparing light intensities with and without the sample.

### Creation of cell–electronics hybrids

#### SWEDs functionalization

SWEDs (5–10 million at a concentration of ~10 million per ml) were immersed in a 10% v/v (3-aminopropyl)triethoxysilane (APTES) solution (Sigma) in 100% ethanol overnight at room temperature. Then these SWEDs were annealed for 2 h at 75 °C to promote the cross-linking of the APTES molecules on PEDOT:PSS. Then, they were rinsed multiple times in deionized water to remove loosely physiosorbed APTES molecules. After this, the APTES-functionalized SWEDs were incubated with equimolar solution of *N*-succinimidyl-4-((5-aza-3,4:7,8-dibenzocyclooct-1-yne)-5-yl)-4-oxobutyrate (Broadpharma) (1 mM solution in PBS-1×) and NHS-Cy3 (1 mM, lumiprobe) for 2 h at room temperature. The SWEDs were constantly stirred during the incubation periods. After the reaction was completed, the SWEDs were rinsed multiple times in PBS-1× and stored at 4 °C until further use.

#### Cell functionalization

Wehi-265.1 cells (American Type Culture Collection) (10–20 million at a concentration of ~2 million per ml) were incubated with succinimidyl 2-azidoacetate (Thermo Scientific) (100 µM in complete cell medium: Dulbecco’s Modified Eagle Medium (Sigma) + 10% fetal bovine serum (American Type Culture Collection) + 1% penicillin streptomycin (PS, Sigma)) solution for 2 h at 37 °C. Thereafter, the cells were rinsed and plated in complete cell medium.

#### Cell–electronics hybrid creation

After the cell functionalization process, the functionalized SWEDs (5–10 million) were incubated with the cells (10–20 million) in complete cell medium (5 ml) to allow the attachment of the SWEDs to the cell membrane for 2 h at 37 °C. Periodic agitation was provided to ensure that the SWEDs did not settle down in the petri dish and to increase the SWED attachment efficiency. For experiments requiring the cells to be fluorescent, NHS-Cy5 (100 µM, Lumiprobe) and Qtracker 705 (QT705, Invitrogen) were added after incubating SWEDs with the cells for 2 h and were allowed to stain the cells according to the manufacturer’s protocol.

#### Cell–electronics hybrid sorting

Cell capture and isolation were performed using FACS. After incubation, a single-cell suspension was created by filtering the solution from a 35-µm nylon mesh and stored on ice. The single cells were loaded onto the sorter (Sony MA900-1) and passed as a stream in droplets, in front of a laser. Cells with SWEDs attached to them showed higher scattering and fluorescence signal (SWEDs were functionalized with NHS-Cy3), and a double gating strategy was used to isolate the cell–electronics hybrids with a purity of 92.4% ± 5.2% (Fig. 3c and Extended Data Fig. 4). When fixing and embedding for imaging, the cells attached to the SWEDs were fixed and embedded on a 35-mm glass-bottom petri dish (no. 1.5). Note that the cells were fixed for imaging purposes only. Live cells were used for i.v. injection. For fixing the cells, 4% paraformaldehyde (PFA, EMS) was added to cells suspended in PBS-1× (cell concentration 2 million per milliliter) in equal volume (to make the final concentration of 1 million per ml) and incubated for 10 min at room temperature. Thereafter, the fixed suspended cells were rinsed with PBS-1× (at least 3 times) to remove any fixing reagent from the solution. The rinsed cells were plated on a poly-l-lysine (Sigma) coated 35 mm glass-bottom petri dish to make them adherent to the glass surface for at least 24 h. Once the cells had adhered to the surface, the PBS-1× solution was aspirated, and the cells were embedded in agarose (5% w/v in deionized water) solution before imaging.

#### Imaging

The cell–electronics hybrids were imaged using a Zeiss Crossbeam 540 SEM and a focused ion beam with a serial sectioning done at 20-nm resolution (Fig. 3a). A FV1200 Olympus confocal microscope was used for fluorescent imaging. Cells were stained with FAM-DBCO dye and SWEDs showed auto-fluorescence owing to P3HT molecules. *Z*-stack images of the hybrids (cells in yellow, SWEDs in green) were taken simultaneously to confirm the SWED attachment to the cell (Fig. 3b).

### Transmigration assay

To determine the stability of cell–electronics hybrids during transmigration across the vascular endothelium, we conducted transmigration assays using a modified Boyden chamber kit (ECM558, Millipore Sigma). The Boyden chamber system is a two-chamber setup with a porous membrane providing an interface between the upper and lower chambers. The upper side of the cell culture insert was coated with fibronectin to support optimal attachment and growth of murine endothelial cells (BALB-5023, Cell Biologics), which were cultured on top of the porous membrane to simulate the endothelial barrier. The integrity of the endothelial monolayer was confirmed by measuring the trans-endothelial electrical resistance using EVOM2 equipment (WPI). The experiment began only when the measured impedance was found to be above 120 Ω cm^2^, ensuring a confluent monolayer of endothelial cells had formed.

To activate the endothelial cells and mimic an inflammatory environment, the cells were treated with TNF at a concentration of 50 ng ml^−1^ overnight. Following the activation of endothelial cells with TNF, each of the suspensions—SWEDs or monocytes or cell–electronics hybrids—were added separately to the upper chamber and were allowed to transmigrate toward a chemoattractant (monocyte chemoattractant protein 1 (MCP-1), 200 ng ml^−1^, Invitrogen) in the lower chamber through the endothelial cell-coated porous membrane. After an incubation period of 48 h, the cells in the lower chamber were quantified using a hemocytometer and confocal microscopy to determine the population and the transmigration rates of monocytes, SWEDs and hybrids and evaluate the stability of the hybrids.

### Subjects used for animal experiments

Male and female Balb/C mice (Taconic) aged 7–12 weeks were maintained with a 12-h light/dark cycle and provided food and water ad libitum, also during the duration of the experiment. Animal husbandry and all experimental procedures were approved by the Massachusetts Institute of Technology Committee on Animal Care.

### Stereotactic injection for the inflammation model

Note that here the stereotactic injection is done only to create the inflammation model. In actual applications of this technology, inflammation will already be present in the target diseased brain regions and our technology does not require any surgery. All mice surgeries were performed under aseptic conditions on a stereotaxic frame. Mice were anesthetized using isoflurane (1–4%). Analgesics were provided after anesthesia: subcutaneous injection of buprenorphine sustained release (1.0 mg kg^−1^) before surgery and lidocaine (0.5%, 2–4 mg kg^−1^) was injected subcutaneously at the incision site before making the incision. Coordinates used for the intracranial injection into the ventrolateral thalamic nucleus region relative to bregma were established according to the Allen Brain Atlas^41^ as follows: anterior–posterior −1.6 mm, medial–lateral +1.0 mm and dorsal–ventral −3.5 mm. A dental drill was used to create an opening in the skull and 1.0 μl of fluorescein isothiocyanate (FITC)-conjugated LPS (5 µg µl^−1^) was injected into the target region. A 10-μl nanofil syringe with a 33-gauge beveled needle was used for the injection. Injection speed of 100 nl min^−1^ was maintained using a micro-syringe pump and its controller. The syringe was left positioned for 10 min inside the brain before injecting LPS as well as before withdrawal from the brain. Skin tissue was closed with an adhesive and sutures, and the mouse was allowed to recover on a heat pad.

### i.v. injection

After the LPS injection, the mice were allowed to recover on a heat pad and transferred to a cage before the next set of experiments. The cell–electronics hybrids solution was prepared using FACS in the meantime for delivering them via i.v. (retro-orbital or tail-vein) injection. Cells were thereafter stained with NHS-Cy5/QT705 (Invitrogen, Thermo Fisher) according to the manufacturer’s protocol. The sorted cell–electronics hybrids were then rinsed and resuspended in PBS at a concentration of 20 million per ml. The final volume of the injection was kept at 100 µl. The entire process was done under sterile conditions. The prepared solution was i.v. injected 6 h after the LPS injection. The mice were allowed to recover after that and kept under observation until they were euthanized.

### Perfusion and imaging

Anesthetized mice were perfused transcardially with 4% PFA in PBS-1× 72 h after the i.v. injection. Brains were harvested and stored in 4% PFA overnight. Then 50-μm thick sections of the brain were sliced coronally using a vibratome (Leica VT1000 S) and imaged using a confocal microscope (Nikon 1AR Ultra-fast confocal microscope) for locating the LPS injection site as well as the distribution of the cell–electronics hybrids. LPS was conjugated with FITC-dye and the cells were stained with NHS-Cy5/QT705 to allow for their simultaneous imaging in separate fluorescent channels.

### Quantifying cell–electronics hybrids in tissue

The quantification of cell–electronics hybrids was carried out by quantifying the content of Ti (as the SWEDs contain Ti layers) using inductively coupled plasma-mass spectrometry (Agilent 7900). Specifically, the brain was digested in nitric acid (70%, trace-metal grade) and hydrogen peroxide (30%, trace-metal grade) in a ratio of 5:1 using UltraWave microwave digestor (Milestone) (Fig. 3g and Supplementary Fig. 11). The entire solution was then diluted with milli-Q water to a final concentration of 3% v/v of nitric acid. One blank and six standards (0, 1 ppb to 100 ppm in steps of 10×) were used for calibration of Ti concentration and ^103^Rh was used as the internal standard during these measurements.

To quantify the number of implanted SWEDs (PEDOT:PSS|PCPDTBT:PCBM|Ti), the Ti content corresponding to a single SWED was calculated. This was based on the amount of Ti present in the SWED (50 nm Ti deposited on a 10-µm diameter device using an electron-beam deposition tool). The intrinsic baseline Ti content in the brain for control animals (without undergoing any administration of SWEDs) was subtracted from the Ti content in the brain for the experimental animals, and the result was divided by the Ti content of a single SWED to estimate the number of implanted SWEDs.

### Assessment of cell–electronics hybrid localization

We used a two-channel imaging system to investigate the localization of self-implanted cell–electronics hybrids in relation to the target region, indicated by LPS image intensity using a logistic regression model (*P*(1|*X*) = 1/exp(−*β*_1_*X* + *β*_0_)). Regions of interest (ROIs) were identified where the cell–electronics hybrids were located. For the actual data from our experiment, 50% of pixels from these ROIs were randomly selected and mapped to corresponding LPS intensities. An equivalent number of pixels from the remaining region, without identified cell electronics, were randomly selected to collectively create the training dataset.

Similarly, the rest of the untrained dataset (50% of pixels from the identified ROIs and an equivalent number of pixels from the remaining region, without identified cell–electronics and not selected in training dataset) were used to create the prediction dataset. For the prediction, a threshold of 0.5 was used for the classification. The final values of the logistic regression analysis (*β*_1,_ *β*_0_ and accuracy) were obtained by averaging the results from 1,000 repetitions of the fitting for each image.

In the control, the mapping between the LPS intensity values and the regions with or without identified cell–electronics hybrids was shuffled. A logistic regression model was then used to evaluate the influence of target region on the localization of cell–electronics hybrids, with the beta coefficient (*β*_1_) and accuracy values to assess the strength of this relationship and the fit of the model, respectively.

To visually compare the experimental and predicted localizations of cell–electronics hybrids, predicted images were generated (Extended Data Fig. 6). The experimental data included an image of FITC-conjugated LPS and an image showing the distribution of self-implanted cell–electronics hybrids. The predicted image was generated using the trained logistic regression model, showing the probability of cell–electronics hybrids localization based on the LPS intensity.

### In vitro patch-clamp experiments

For our in vitro electrophysiology experiments, we used a carefully designed protocol to investigate the interaction between SWEDs and neurons. Hippocampal neuron cultures (grown on glass coverslips) were transferred to glass-bottomed petri dishes containing fresh media and placed on an inverted microscope (Nikon Ti2-E) equipped for patch-clamp recordings and imaging after 14 days in vitro. Released SWEDs were drop-casted in the culture and neurons with SWED on top of them were identified for patching using an inverted microscope.

Patch pipettes (4–5 MΩ resistance) were prepared using a micropipette puller (P-1000, Sutter). The intracellular solution for whole-cell recordings consisted of K-gluconate, NaCl, CaCl_2_, MgCl_2_, EGTA, HEPES, Mg-ATP and Na-ATP, with pH and osmolarity adjusted to physiological levels. Voltage-clamp and current-clamp recordings were conducted using a Multiclamp amplifier and digitized for analysis (Molecular Devices). Optical illumination was achieved using a diode laser (Doric) positioned on top of the targeted neuron interfacing with the SWED. Light control and synchronization with electrophysiology recordings were managed through custom software and hardware triggers. The generated action potentials show precise temporal correlation to optical pulse offset with a consistent latency of 174 ms ± 52 ms (Extended Data Fig. 7b,c). In addition, the observed neural response pattern (as shown in Extended Data Fig. 7) of initial hyperpolarization followed by depolarization leading to action potentials aligns with established literature on capacitive neuron-device coupling^42,43^ and postinhibitory rebound mechanism^44^, while the TiN interface properties likely contribute to the observed stimulation efficacy^45^.

### In vivo c-Fos modulation and analysis

In this study, 18 mice were allocated into two distinct groups to investigate the effects of self-implanted hybrids, monocytes and NIR light on neural activation. The first group, comprising nine mice, received i.v. injections of cell–electronics hybrids while the second group (nine mice) received i.v. injections of cells (without the SWEDs). After a recovery period of 72 h, 4 mice from the first group and 5 mice from the second group were randomly selected for optical stimulation. The selected mice were anesthetized following previously established protocols, with their heads securely fixed. Optical stimulation was administered using a 792 nm wavelength laser (HJ Optronics), delivering a pulse sequence of 15 mW mm^−2^ intensity, 10-ms pulse width, at a frequency of 20 Hz for a duration of 20 min. To allow for adequate c-Fos protein induction, these mice remained anesthetized for an additional 90 min poststimulation before being euthanized. The remaining mice from the two groups did not receive optical stimulation but were subjected to the same conditions of housing, habituation and a 90-min anesthetization period before euthanasia.

After perfusion, brain was extracted and postfixed in 4% PFA overnight for all the animals. Coronal brain sections (50 µm) were prepared using a vibratome (Leica VT1000 S) and stored in PBS-1×. For IHC, tissues were incubated overnight with polyclonal rabbit antibodies raised against c-Fos protein (1:500, ABE457, sigma). c-Fos-positive cells were visualized using immunofluorescence with donkey anti-rabbit Alexa Fluor 647 (1:500, Thermo Fisher; 2 h of incubation at room temperature). All the cell nuclei were stained with Hoechst (Invitrogen). The stained slices were imaged using a slide scanner (TissueFAXS SL) and a confocal microscope (Nikon Ti, CSU-X1 confocal module) with a ×40 objective. For c-Fos-positive cell counting and quantification, an initial mask was drawn by a researcher who was blinded to the experiments, to delineate the target region (characterized by nuclei pattern). The mask was then extended by 50 µm in all directions to account for activation of neighboring neurons. Cell Profiler software was used to identify all c-Fos positive cells within the masked region, and results were expressed as cells per square millimeter (Fig. 4f). An adaptive thresholding approach based on the Otsu algorithm was implemented to segment the cells, with a c-Fos-positive cell being defined as having a mean intensity of the ROI 2.25 times greater than the average background of the entire image and confirmed by colocalization with the cell nucleus.

For the radial distribution of c-Fos positive cells, an initial mask was used to delineate the target region boundary. Then, polygonal ring masks (based on this boundary shape) with 20-µm radial width and varying radial distances were generated using MATLAB. Cell Profiler software with an adaptive Otsu algorithm, was used for cell identification as described above and any ROIs from c-Fos positive cells within these rings were counted and reported per unit area. The distribution of these cells was plotted as a function of radial distance with zero being at the boundary of the target region and positive and negative values corresponding to regions outside and inside the target, respectively (Fig. 4g).

### Single-unit recording

Mice with or without self-implanted cell-electronic hybrids (or monocytes) in the ventrolateral thalamic nucleus region were initially sedated with 3% isoflurane in a mixture of air, and subsequently maintained anesthetized by 1–2% isoflurane after being immobilized in a stereotactic frame. Body temperature was maintained at 37 °C with a heat pad. The epicranium was incised to expose the skull. A craniotomy, 1 mm in size was performed at 1.60 mm posterior to the bregma and 1.00 mm right of the midline (at the LPS injection site). Commercially available 4 × 4 matrix electrodes (50 µm in diameter, impedance ranging between 0.5 MΩ and 2 MΩ) with 200-µm spacing were used to confine the recording site to 800 µm × 800 µm. The electrode array was stereotactically implanted vertically at the inflamed region with the tips of the recording electrodes extending 3.3 mm to 3.7 mm into the brain tissue from the surface. Recorded signals were monitored with Doric Neuroscience Studio V6. Action potentials from electrophysiological signals were amplified 2,000 times with high-pass filtering at 0.25 kHz. The resultant waveform was sampled at 30 kHz and low-amplitude optical artifacts coinciding with pulse onset and offset were systematically removed using automated detection and filtering protocols (Supplementary Fig. 12). Thereafter, single units (signal-to-noise ratio ≥4) were identified by principal component-based spike sorting, where a transient excitatory response was counted if the unit activity exceeded the 99% confidence interval (*Z* value > 2.33) in 2 consecutive bins (bin size, 100 ms) in the experimental condition (0–2 s after stimulus onset) in the normalized peri-event histogram and, when compared with baseline activity, the maximum firing rate increased in more than 50% of the trials. Optical stimulation was delivered using a NIR laser pulse (792 nm wavelength, 100 ms duration, 15 mW mm^−2^) every 10 s during recordings. These optical parameters induced minimal tissue temperature changes, as demonstrated by heat diffusion simulation studies (Supplementary Fig. 13).

For our methodology, we found that 14 out of 64 units showed temporally consistent activation patterns following optical stimulation. These active units exhibited temporally consistent responses correlated with optical pulse timing (within hundreds of milliseconds after optical pulse offset (Fig. 4h and Supplementary Fig. 3)) as seen from the pooled *z*-score plot (Fig. 4i), while nonactive units showed no temporal relationship with stimulation parameters (Supplementary Fig. 14). This 22% sampling efficiency compares favorably with FDA-approved neuromodulation technologies such as repetitive transcranial magnetic stimulation^46^, which demonstrates 28% efficiency in single-unit activation studies.

### Statistical analysis of spike timing and time locking

To evaluate the likelihood that the short first spike timing from the optical pulse offset observed in our data occurred by chance, we generated 10,000 control datasets, in which the *t* = 0 event of each trial was randomly reassigned to a time point within the baseline period, that is, *t* = −3 s to t = 0 s of that trial. Note that *t* = 0 in the original trial marks the onset of the laser pulse. This randomization approach creates a null distribution that helps determine whether the observed neuronal responses are truly linked to the optical stimulation rather than occurring by random chance.

We determined the percentile rank of the observed median timing of the first spike timing from the optical pulse offset within the distribution of median timings from the control datasets. This percentile rank provided a statistical measure of how likely the observed shorter spike timing could have occurred by chance. Similarly, to evaluate the temporal consistency, we determined the percentile rank of the observed MAD of the first spike timing from the optical pulse offset within the distribution of MADs of the timings from the control datasets. The use of MAD provided a robust measure of variability that is less sensitive to outliers than the standard deviation, allowing us to quantify how consistently neurons respond to the optical stimulation.

### Imaging for electrode mapping with hybrid localization

To assess the spatial and functional relationship between electrodes, recorded single units and self-implanted cell–electronics hybrids, we used a combination of electrode prestaining and postexperiment IHC. Electrodes were stained with (1,1′-dioctadecyl-3,3,3′,3′-tetramethylindocarbocyanine perchlorate) (DiI dye, 2% w/v in DMSO) before insertion. Following single-unit recording experiments, brains were extracted and postfixed in 4% PFA overnight. Axial brain sections (100 µm) were prepared using a vibratome (Leica VT1000 S) and stored in PBS-1×. The sliced brain tissues were then analyzed to correlate electrode proximity to cell–electronics hybrids with recorded neuronal activity. The histological analysis combined with electrophysiological recordings (Supplementary Fig. 15) demonstrated that only neurons adjacent to cell–electronics hybrids responded to optical stimulation, while neurons recorded from electrodes positioned farther away remained unresponsive, confirming spatial precision of neuromodulation.

### Cytotoxicity assay for monocytes

Colorimetric MTT assays were performed in 96-well plates where SWEDs at various concentrations (10–10,000 SWEDs per µl) were added to each well plated with the cells (2–4 million per ml). After each time interval (Supplementary Fig. 5), 10 μl of MTT was added to the medium and incubated for 4 h. Then 200 μl of DMSO was added and mixed in each well. The absorbance signal was measured using a spectrophotometer (Spark, Tecan) at 570 nm and the background (measured at 630 nm) was subtracted from the signal to obtain the normalized values.

### Cytotoxicity assay for cultured neurons

E18 Sprague Dawley rat dissociated hippocampal neurons were purchased from Brainbits. Neurons were cultured in the 96-well plates. The well plates were coated with 50 µl of poly-d-lysine (100 µg ml^−1^) to promote cell adhesion. Neurons were plated at a concentration of 10^4^ per well and allowed to grow for 7–10 days. Then, 50 µl of SWEDs at various concentrations (10^5^ to 10^7^ ml^−1^) were incubated for the studied time intervals (Supplementary Fig. 6). Thereafter, 10 μl of MTT was added to the medium and incubated for 4 h. Then 200 μl of DMSO was added and mixed in each well. The absorbance signal was measured using a spectrophotometer (Spark, Tecan) at 570 nm and the background (measured at 630 nm) was subtracted from the signal to obtain the normalized values.

### Blood count and blood serum chemistry analysis and histology

Blood samples were collected at two distinct time points (day 3 and day 12 after LPS injection). For the complete blood count analysis, 6 drops of blood from the facial vein pricked by a 21-G needle were collected in an EDTA-lined tube (20.1278.100, Sarstedt) and mixed by inverting back and forth on a rocker and submitted immediately for analysis. Before transcardial perfusion, 400 µl of blood was collected via cardiac puncture and stored in serum separator tubes (BDAM367985, VWR) for serum analysis. The blood was allowed to coagulate at room temperature for 15 min and spun down at 2,000 rpm for 10 min. The serum was collected and stored at −20 °C until analysis.

After transcardial perfusion, tissue samples of the major organs were collected for histological examination. The tissue samples were fixed, paraffinized, sectioned, H&E stained and scanned using a digital whole slide scanner (Aperio).

### OFT

The OFT was conducted (day 2 and day 11 after LPS injection) to assess the locomotor activity of the mice. After habituation for 10 min individually in the home cage in the behavior testing room, each mouse was individually placed in the center of an OF arena of size 400 mm × 400 mm × 300 mm and allowed to explore freely for 10 min. A 10-min behavior recording was started right after the mouse was place in the OF arena. Locomotion was recorded two-dimensionally at 10 Hz from top-view with a CCD video camera installed above the center of the OF arena. Right after recording, the mouse was placed back in its home cage, and returned to the holding room. Video image data were processed using a custom script written in MATLAB R2023b (MathWorks).

### NORT

The NORT was performed (day 3 and day 12 after LPS injection) to evaluate the recognition memory of the mice. The test consisted of a 10-min training phase, where the mice were exposed to 2 identical objects, and a 5-min testing phase, where 1 of the familiar objects was replaced with a novel object, with a 1-h interval between the 2 phases. Right after recording, the mouse was placed back in its home cage, and returned to the holding room. The time spent exploring the novel object versus the familiar object was analyzed using custom-written MATLAB scripts. The discrimination index was defined as *t*_new_/(*t*_new_ + *t*_old_), where *t*_new_ and *t*_old_ are the times spent exploring the new and old objects, respectively.

### Testing immunoreaction to SWEDs

To examine potential immunoreaction to SWEDs, eliminating the immune reactions stemming from LPS (which is used to create the inflammation model), SWEDs were directly injected intracranially into the brain (instead of the self-implantation procedure). Intracranial injection of PBS under identical conditions was used as a control. SWEDs (at a concentration of 10 million per ml) or PBS (control) were unilaterally injected (2 μl) using a glass pipette into the mouse brain at the following coordinates: anterior–posterior −1.6 mm, medial–lateral 1 mm and dorsal–ventral −2.5 mm. Mice were euthanized at 1 day, 3 days and 7 days postinjection (*n* = 3 mice for each time point). Brains were extracted, coronally sectioned (50 μm) and immunohistochemically (IHC) stained for astrocyte marker GFAP (1:500, Thermo Fisher) and microglia marker-ionized calcium-binding adapter molecule 1 (Iba-1) (1:500, SYSY) following the manufacturer’s protocol. GFAP and Iba-1 IHC used goat anti-rat Alexa Fluor 488 (1:500, Biotium) and donkey anti-guinea pig Alexa Fluor 633 (1:500, Biotium), respectively, for secondary antibody staining. Hoechst (1:10,000, Thermo Fisher) was used to counterstain labeled cell nuclei.

Confocal imaging of stained sections was done using a Nikon Ti microscope (CSU-X1 confocal module). The SWEDs or PBS injection sites were delineated using a MATLAB script. Cell Profiler was used to quantify GFAP and Iba-1 expression based on fluorescence intensity within the defined injection region. Measurements were averaged across three sections per animal. SWEDs and PBS groups were statistically compared at each time point.

### Clearance studies of i.v. injected cell–electronics hybrids

The clearance kinetics of the injected cell–electronics hybrids were investigated using the IVIS Spectrum imaging equipment (PerkinElmer). The mice with the LPS injection in the brain were i.v. injected with the fluorescently labeled cell–electronics hybrids. At various time points after injection, the mice were anesthetized with isoflurane (3% in air) and the fluorescence intensity in the animal body was examined under the IVIS system to track the clearance of the cell-electronic hybrids. Once clearance of the cell–electronics hybrids was confirmed (fluorescence intensity returning to baseline) under in vivo imaging, the mice were euthanized by transcardial perfusion and major organs (kidneys, spleen, heart, brain, liver and lungs) were harvested. These organs were subsequently imaged ex vivo under the IVIS system to provide additional confirmation regarding the clearance.

### Continuous health monitoring

Throughout the study, the health of the animals was continuously monitored. This included regular checks on their body condition score, a widely accepted method to assess the overall health status of an animal. The body condition score provides a visual assessment of an animal’s muscle and fat, which can indicate whether the animal is underweight, overweight or at an ideal weight.

In addition to body condition scoring, the water intake and the body weight of the animals was also tracked. Changes in water consumption can be an early indicator of health issues. Similarly, changes in body weight can signal potential health problems.

### H&E studies of brain tissues

To understand whether the SWEDs cause any adverse effect on brain tissue (eliminating any influence of LPS, which is used to create the inflammation model as well as to study chronic effects), SWEDs (10 µm diameter, 5 µl of solution at a concentration of 10 million SWEDs per ml) were directly injected stereotactically into the hippocampal region (anterior–posterior −2 mm, medial–lateral 1 mm, dorsal–ventral −2 mm). The animals were euthanized via transcardial perfusion in 4% v/v of PFA in PBS-1× at 1 day, 1 week, 1 month and 6 months postinjection. The brain was isolated and stored in 4% PFA (in PBS-1×) overnight before slicing it using a vibratome (Leica VT1000 S). Automated stainer (Tissue-Tek Prisma Plus) was used for staining the samples with H&E and slides were imaged using a digital whole slide scanner (Aperio) at ×40.

### Biphasic pulse generator

Simulations were performed using Cadence Virtuoso and Spectre. Experimentally measured current–voltage characteristics of 10-μm diameter P3HT (D_1_) and PCPDTBT (D_2_) SWEDs when embedded in the brain tissue at a depth of 0.5 mm and wirelessly controlled with 520-nm and 785-nm illumination (Fig. 2h), were fitted using third-order polynomials. The polynomial fits were used to model the operation of D_1_ and D_2_ in Supplementary Fig. 16 using a Verilog-A script. Metal-oxide-semiconductor field effect transistors M_1_ and M_2_ were modeled using BSIMSOI v.4.7.1 with gate length *L* = 45 nm, gate width *W* = 450 nm, buried oxide thickness *T*_BOX_ = 25 nm, top silicon thickness *T*_Si_ = 10 nm and gate dielectric thickness *T*_ox_ = 2 nm, as fully depleted silicon on insulator field effect transistors. The complementary metal-oxide-semiconductor footprint for the designed circuit was <1 μm^2^ (Supplementary Fig. 17). The load was modeled based on the electrode–electrolyte impedance of 2-μm diameter raised Pt-electrodes in PBS-1× (ref. ^47^), with series parasitic resistance *R*_p_ = 1.02 MΩ and pseudo-capacitance *C*_P_ = 74.4 pF per electrode. Temperature was set at 37 °C to account for operation of the circuit in vivo.

The operating points of metal-oxide-semiconductor field effect transistors M_1_ and M_2_ were set self-consistently in the subthreshold regime based on the operating points of the driving SWEDs. When actuated by a 785-nm (520 nm) laser pulse, D_2_ (D_1_) developed a larger voltage than D_1_ (D_2_), which appeared as the gate-source voltage (*V*_GS_) for M_2_ (M_1_). For M_1_ and M_2_ operating in the subthreshold regime, a relatively small difference in *V*_GS_ resulted in a large difference between their drive currents. Thus, M_2_ (M_1_) was selectively turned on and current flowed from D_2_ (D_1_) to the load and back through M_2_ (M_1_). We created a negative pulse of 65 μs (ref. ^48^), a gap (no illumination) of 50 μs followed by a charge balancing, 190-μs positive pulse by remotely controlling the illumination timings. The frequency of operation was chosen to be 200 Hz.

### Reporting summary

Further information on research design is available in the Nature Portfolio Reporting Summary linked to this article.

## Online content

Any methods, additional references, Nature Portfolio reporting summaries, source data, extended data, supplementary information, acknowledgements, peer review information; details of author contributions and competing interests; and statements of data and code availability are available at 10.1038/s41587-025-02809-3.

## Supplementary information

Supplementary Information Supplementary Figs. 1–18, Tables 1–4, Note and Refs.

Reporting Summary

## Source data

Source Data Figs. 2–5 and Extended Data Figs 1–3, 5, 7, 9 and 10. Statistical source data.

## Data Availability

All data needed to evaluate the conclusions in the paper are present in the paper, Supplementary Information and/or via DRYAD at 10.5061/dryad.pzgmsbd12 (ref. ^49^). Source data are provided with this paper.

## Code availability

Custom codes corresponding to different analysis can be found via DRYAD at 10.5061/dryad.pzgmsbd12 (ref. ^49^).
