# Supplementary material for: A nonsurgical brain implant enabled through a cell–electronics hybrid for focal neuromodulation
Source: Nat Biotechnol. 2025 Nov 5;44(8):1329–39. doi: 10.1038/s41587-025-02809-3 (PMC13461362; doi:10.1038/s41587-025-02809-3)
Supplement: Supplementary file 1 [file 41587_2025_2809_MOESM1_ESM.pdf]

nature biotechnology

Supplementary information https://doi.org/10.1038/s41587-025-02809-3

A nonsurgical brain implant enabled through a cell–electronics hybrid for focal neuromodulation

In the format provided by the authors and unedited

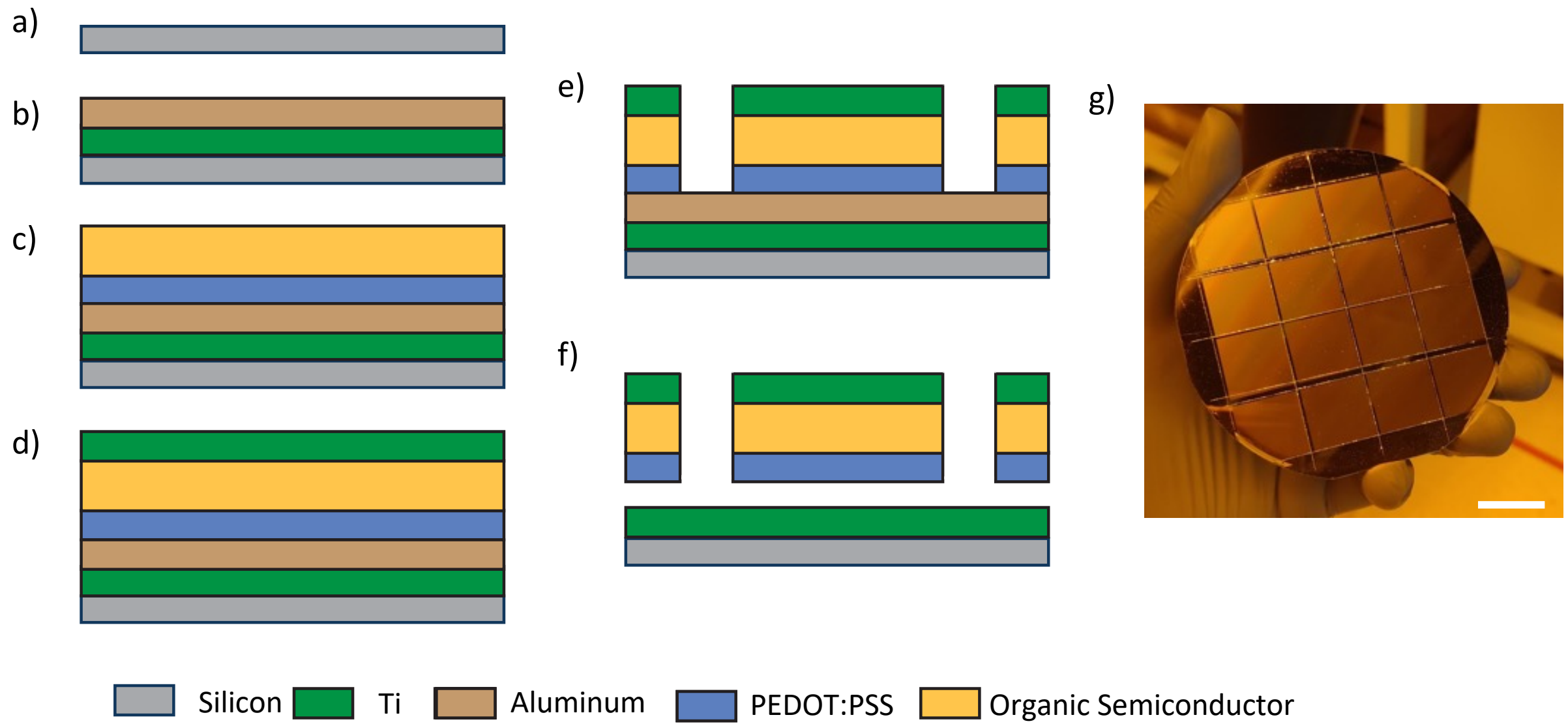

Supplementary Data Fig. S1. Fabrication process

(a) Si wafer was used as the starting substrate. **(b)** A thin film of Ti/Al (100 nm/200 nm) was deposited using electron-beam evaporation to serve as the sacrificial layer for lifting off the devices from the substrate. **(c)** Spin coating of organic polymers (PEDOT:PSS (~50 nm) – in air and binary blend of organic semiconductor (~100 nm) – P3HT:PCBM or PCPDTBT:PCBM – inside the glovebox). **(d)** Deposition of a thin film of Ti (50 nm) using electron-beam evaporation. **(e)** Patterning the micron-sized devices using photolithography and dry-etching techniques. A 1 µm thick photoresist (AZ3312) was spin-coated on top of Ti (top layer), the patterns were made using MLA-150 (mask-less photopatterning) and thereafter the photoresist was allowed to hard-bake before dry-etching**.** For dry-etching, SF_6 based chemistry was employed to selectively etch exposed Ti metal. After this, oxygen plasma was used to etch away the organic polymers (including the photoresist). For this step, Ti served as a hard-mask for the polymer stack underneath. **(f)** For releasing the devices from the Si substrate, the exposed Al layer underneath was allowed to react with diluted TMAH solution (2.7% v/v in water) and the released devices were collected and stored in DI water. **(g)** Devices post-fabrication on a 4-inch silicon wafer. Scale bar – 20 mm.

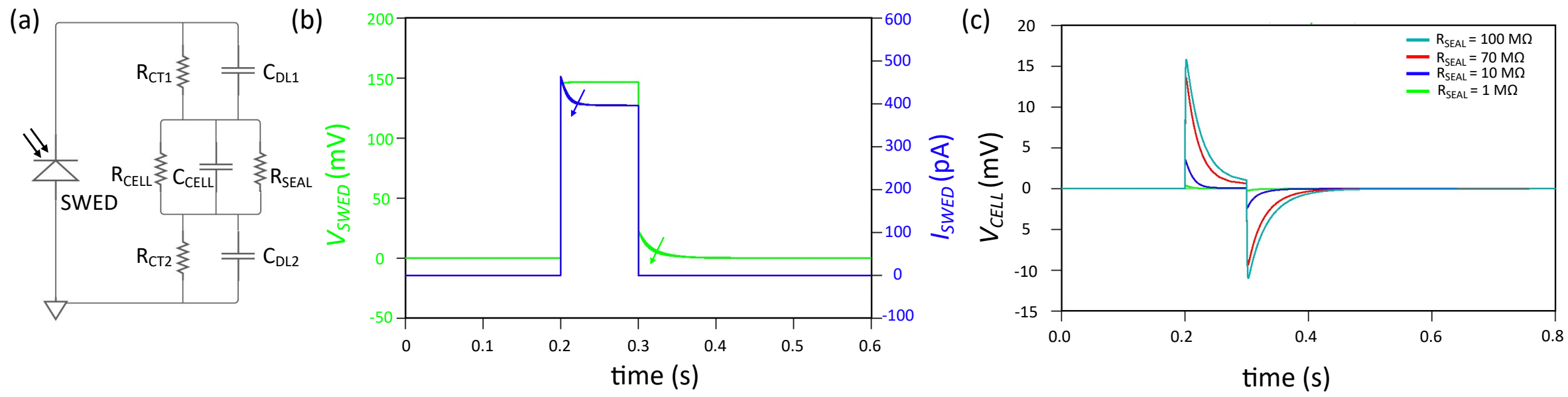

Supplementary Data Fig. S2. SWED's operation in the extracellular environment

(a) Schematic circuit diagram for the SWED characterization in free-floating form in the extracellular environment. Here, $R_{CT1,2}/C_{DL1,2}$ - interface impedance of the two electrodes (PEDOT:PSS, TiN) of the SWED, R_{CELL}/C_{CELL} - impedance of the cell membrane and R_{SEAL}- leakage impedance path for the charges generated by the SWED. **(b)** Voltage and current generated by the SWED as a function of R_{SEAL}. Arrows point towards increasing values of R_{SEAL} (1 KΩ to 100 MΩ). Note: The operating point remains stable irrespective of the R_{SEAL} value. **(c)** Transmembrane potential induced by the SWEDs on the neural cell membrane as a function of R_{SEAL}. SWED was turned ON at 0.2 s for 100 ms for (b) and (c).

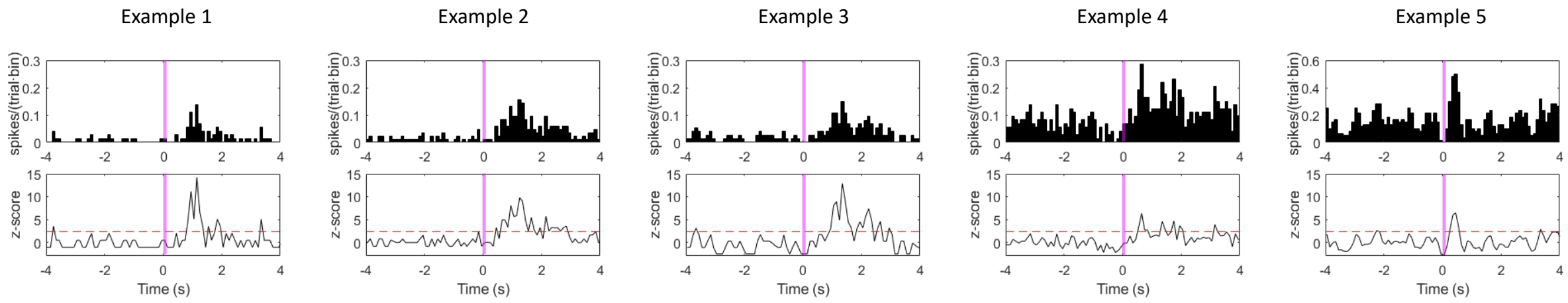

Supplementary Data Fig. S3: Example datasets from multichannel single-unit recordings showcasing histogram and the z-score plots for the experimental group (Hybrids + NIR Light). Vertical shaded line - time = 0-100ms for the optical pulse. Horizontal dashed line (in z-score plot) – z=2.33, bin = 100ms, Optical pulse – 100 ms, 15 mW/mm^2, 792 nm.

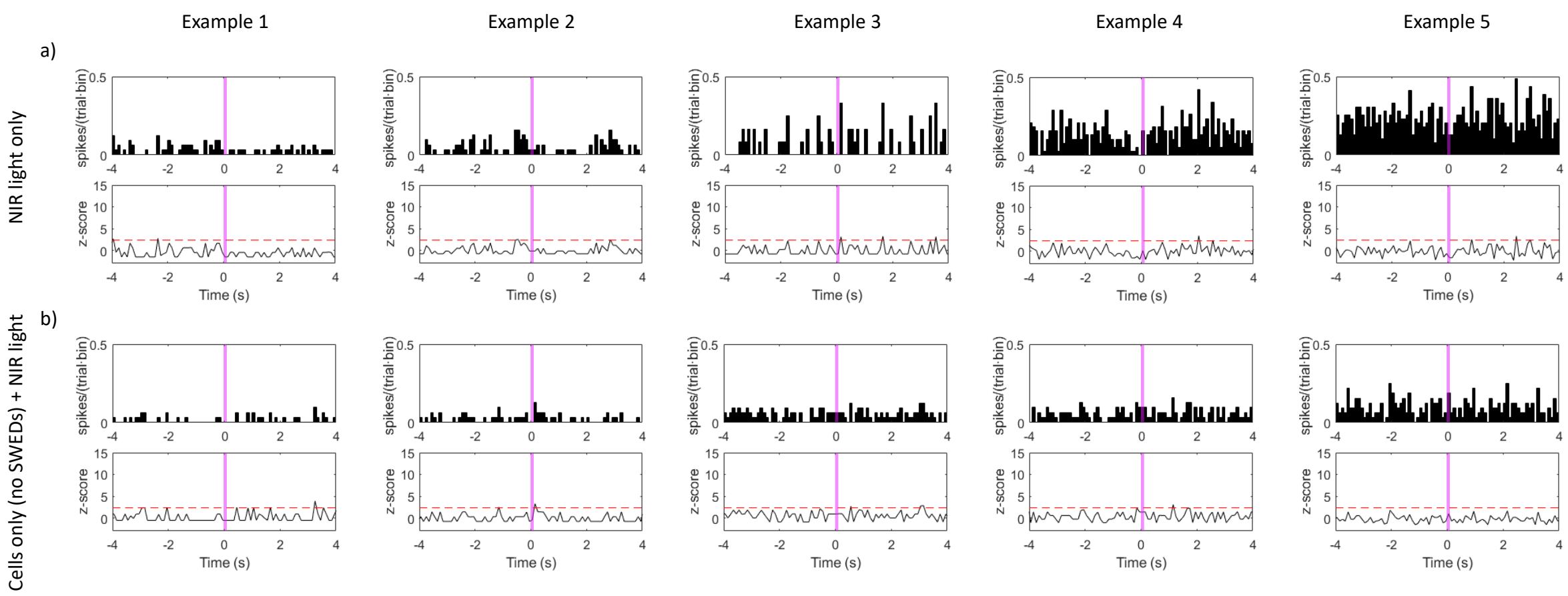

Supplementary Data Fig. S4: Example datasets from multichannel single-unit recordings showcasing histogram and the z-score plots for the control datasets: **(a)** NIR light only and **(b)** cells (without SWEDs) + NIR light. Vertical shaded line - time = 0-100ms for the optical pulse. Horizontal dashed line (in z-score plot) – z=2.33, bin = 100ms, Optical pulse – 100 ms, 15 mW/mm^2, 792 nm.

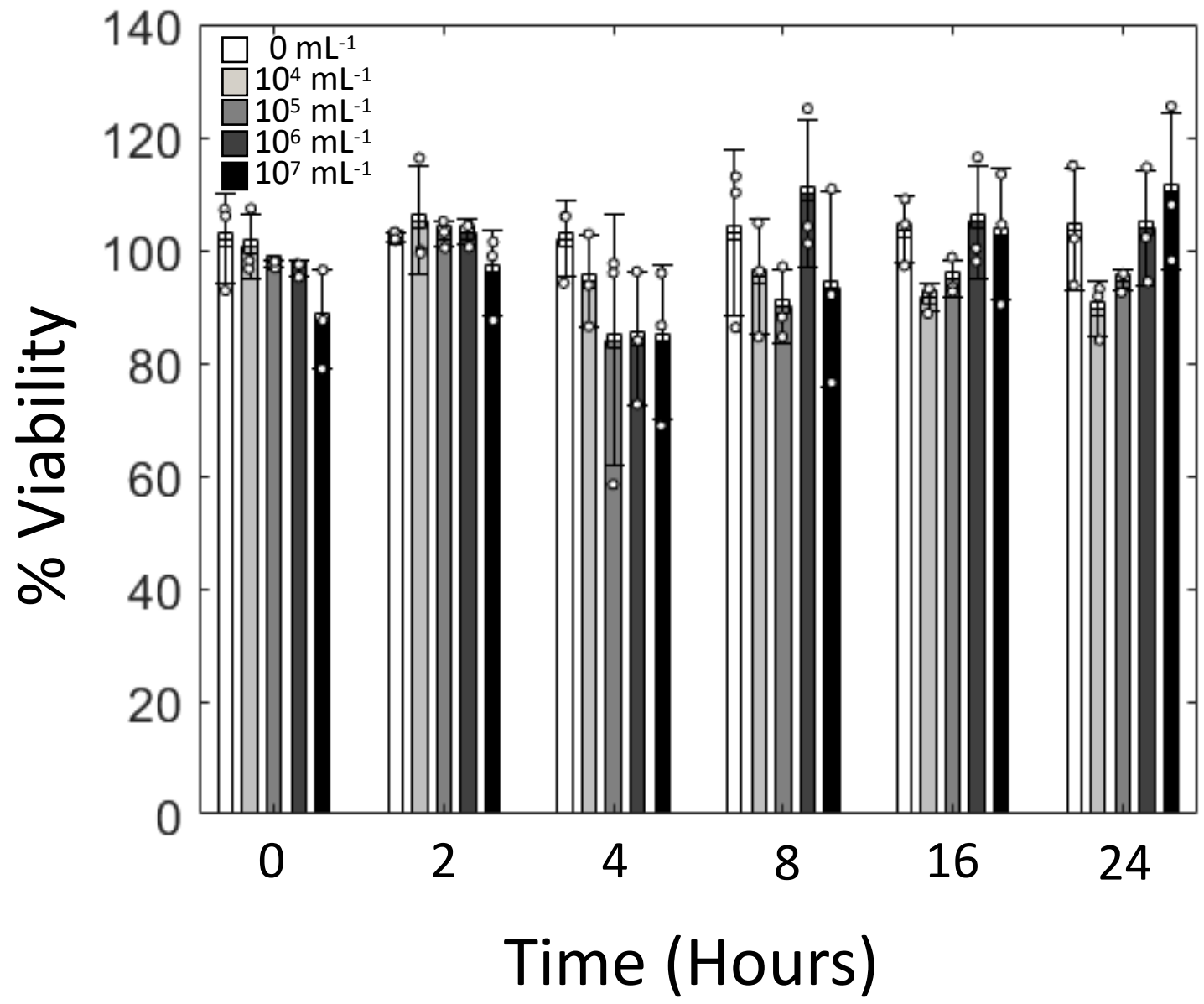

Supplementary Data Fig. S5. MTT assay for cytotoxicity in monocytes

Quantitative toxicity analysis of SWEDs (10 µm in diameter) incubated with wehi-265.1 monocytes. Time varying MTT assay showing biocompatibility of these SWEDs with minimal toxicity to cells. The x-axis represents the incubation time of the DBCO-functionalized SWEDs with Azide-functionalized monocytes. The concentration of SWEDs used for the toxicity assessment was varied between 10^4 to 10^7 mL^{-1} and the concentration of cells used was 2 million/mL. Note that, for intravenous injection, SWEDs at a concentration of 10^6 mL^{-1} were incubated with cells (2-4 million/mL) for 2 hours. Values represent median $\pm$ SD (n=3 independent experiments).

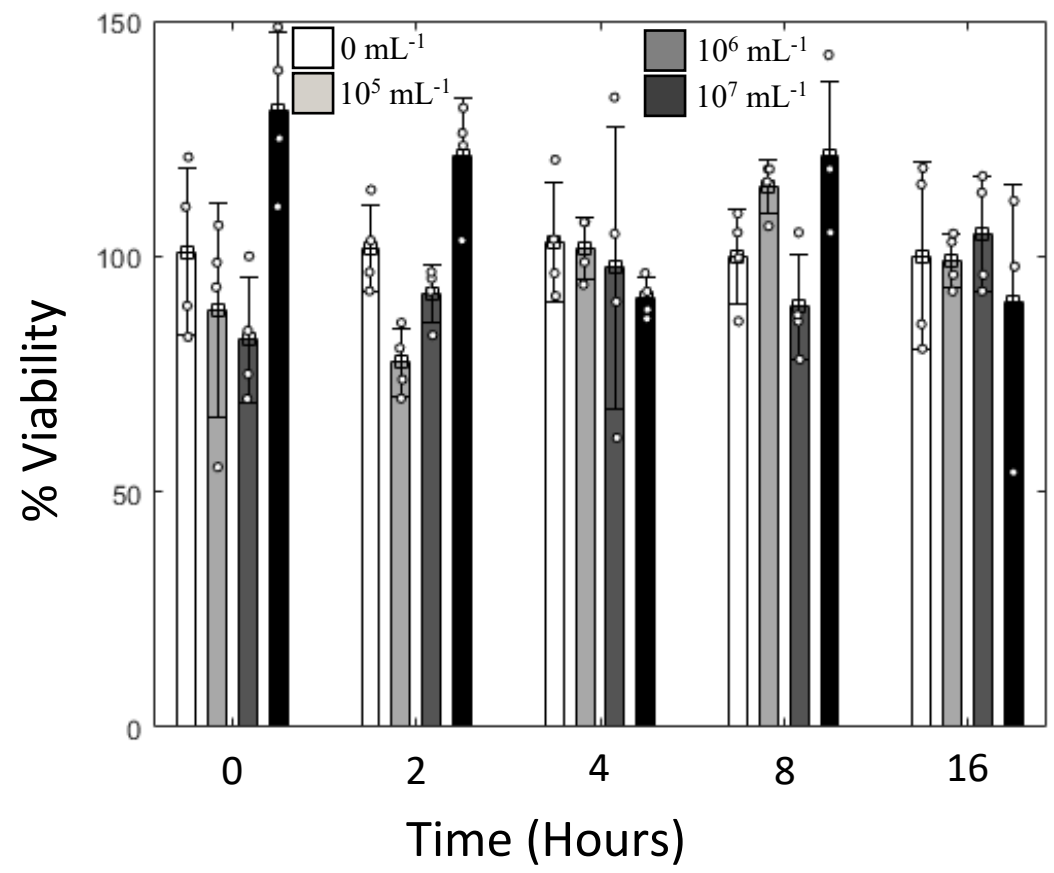

Supplementary Data Fig. S6. MTT assay for cytotoxicity analysis in neurons: Quantitative toxicity analysis using MTT assay for subcellular sized SWEDs (10 µm in diameter) incubated with cultured neurons. The plot demonstrates the biocompatibility of these SWEDs with neurons over a period of 24 hours. 50 µl of SWEDs at various concentrations (10^5 to 10^7 mL^{-1}) were incubated with neurons cultured in a 96 well plate for 7-10 days prior to the experiment; values represent mean ± SD (n=3 independent experiments).

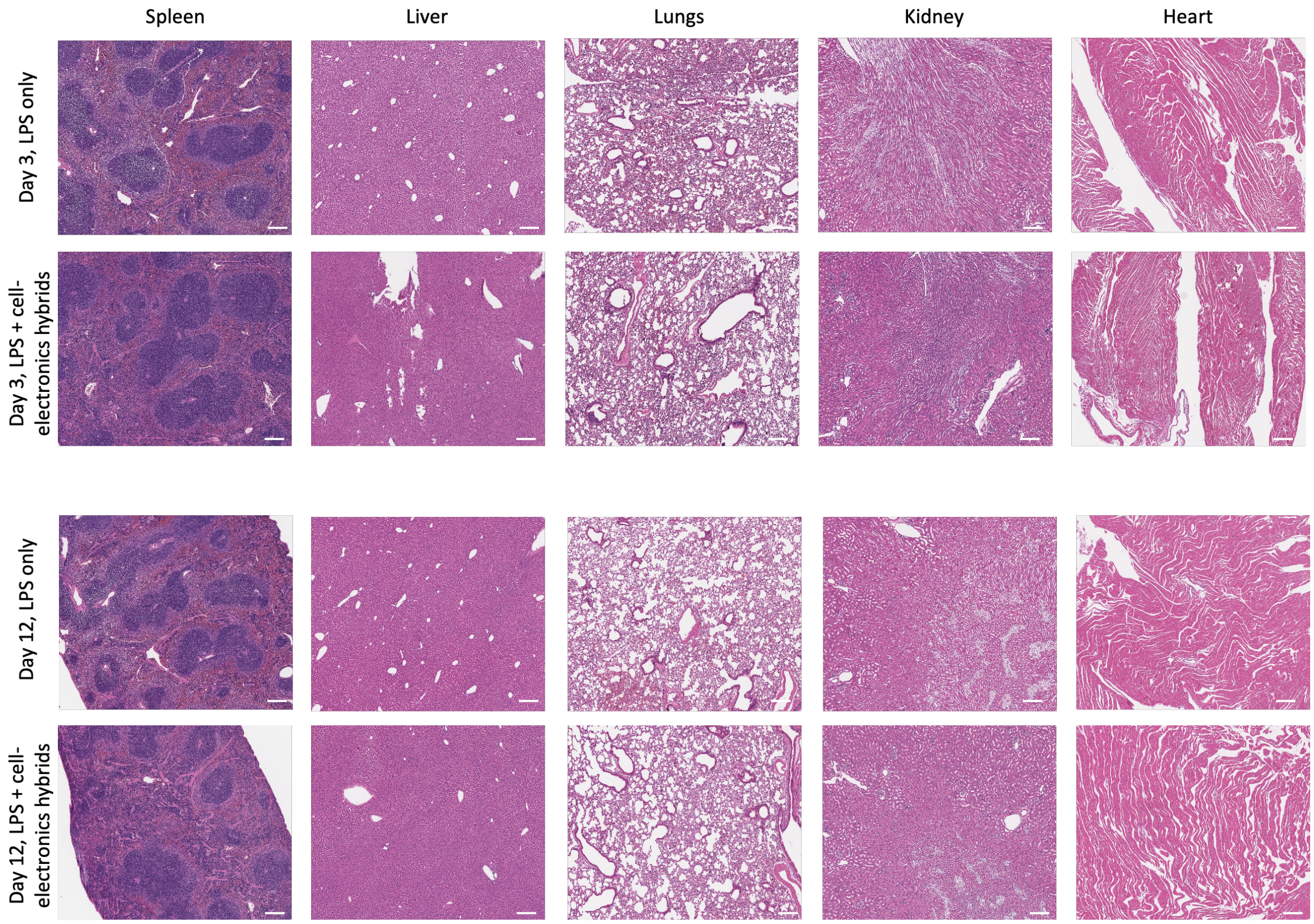

Supplementary Data Fig. S7. H&E staining: Representative histology (H&E) of the mice organs 3 days and 12 days post LPS injection in the brain. Experimental group underwent administration of cell-electronics hybrids (2 million) via intravenous injection while controls did not (n=3 mice). Scale bar - 200 μm.

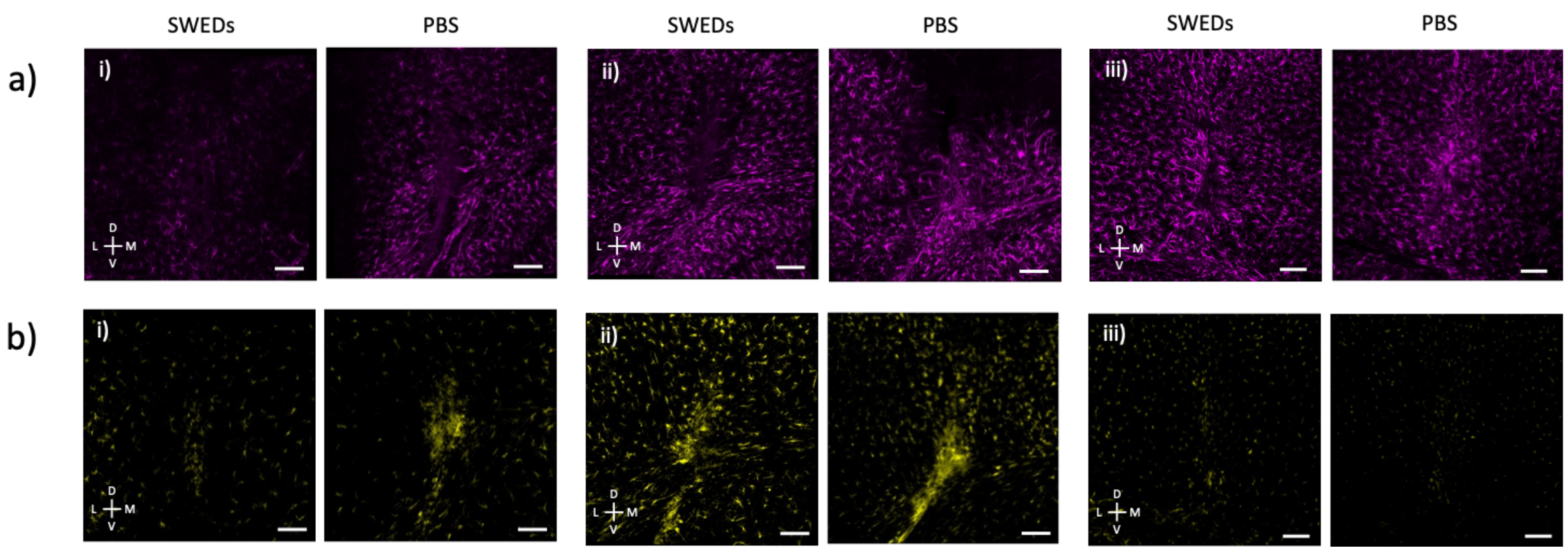

Supplementary Data Fig. S8. Investigation of potential immunoreactions to SWEDs. To differentiate potential immunoreactions to SWEDs from that to LPS (which create strong immunoreactions by itself) in the brain tissue, SWEDs were intracranially injected in this study. The intracranial injection of PBS was used as control. **(a)** GFAP and **(b)** Iba-1 immunohistochemical staining of brain tissue (i) 1 day, (ii) 3 days, and (iii) 7 days after intracranial injections of SWEDs or PBS (control) (n=3 mice). Scale bar - 100 µm.

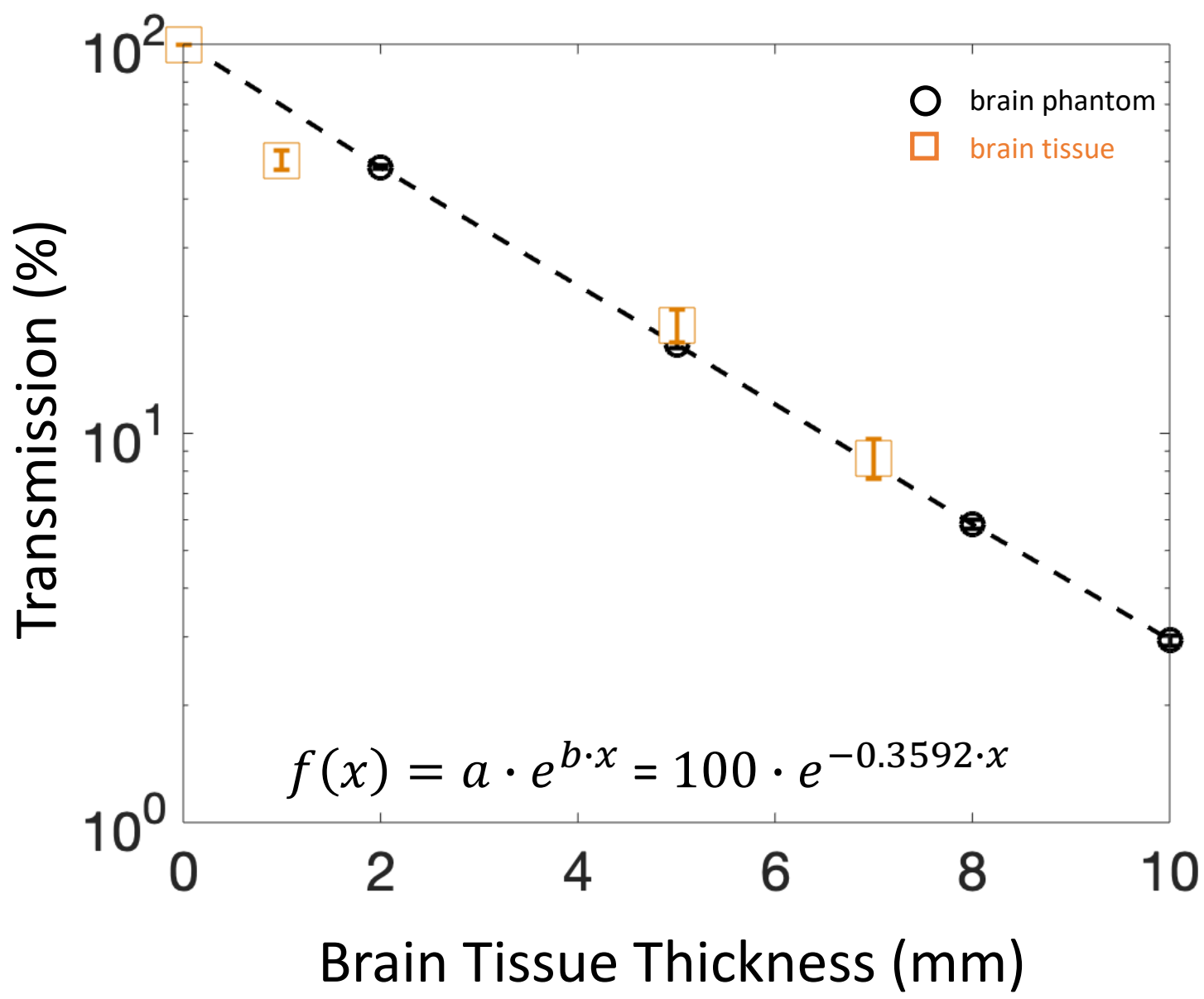

Supplementary Data Fig. S9. Near-infrared light transmittance in brain tissue
Transmission intensity of near-infrared (792 nm, HJ Optronics) light measured at various thicknesses of the brain tissue and the brain phantom (see **Methods 6**). The experimental data is shown as discrete points with error bars. Based on exponential curve fitting (Beer-Lambert Law), the expected transmission trend has been plotted as a dotted line. For the curve fitting, *x* corresponds to the brain tissue thickness and has the units of mm. At least three brain slices (for each thickness) were used from two different animals. The plot represents median $\pm$ SD (n=3 independent measurements). Note: while these measurements are specific to the optical system utilized in our study, previous research[1] has demonstrated that NIR light can penetrate even 3 cm into a human head with intact skull with as high as 2.9% transmission, highlighting the significant potential of NIR light for deep tissue applications. In addition to transcranial approaches, alternative methods such as intranasal, transauricular (through the ear canals), and transoral delivery have been discussed as viable routes for delivering light to deep brain regions closer to these sites, which can further enhance the transmission of significant NIR light to the diseased regions[2].

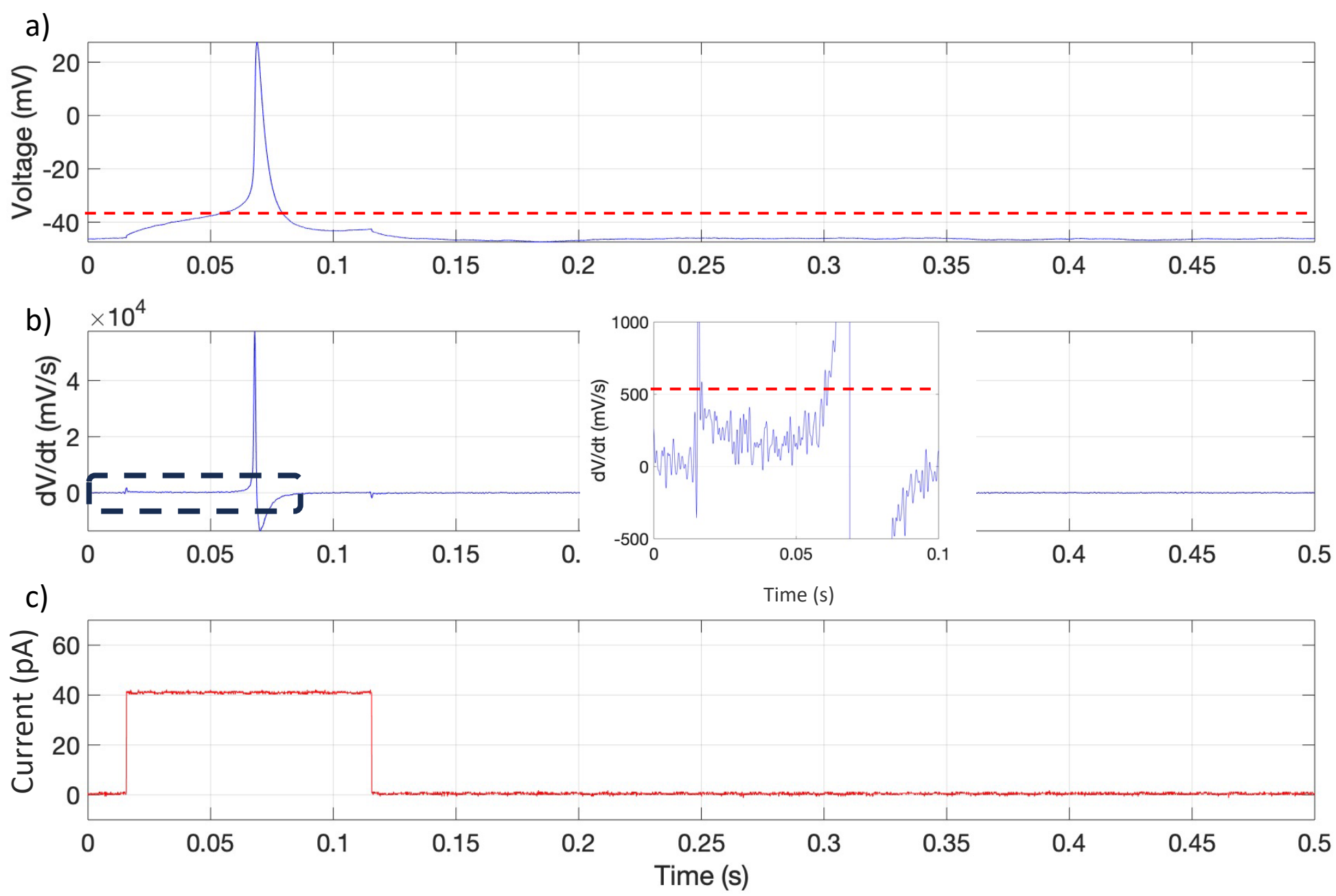

Supplementary Data Fig. S10. Threshold voltage estimation for neurons

(a) – (c) Whole-cell current clamp recording of a representative primary embryonic rat hippocampal neuron cultured in-vitro. **(a)** presents the transmembrane potential V_M, **(b)** presents the time derivative plot of the recorded transmembrane potential (inset shows the zoomed-in image (of the square box) showing the time derivative of the transmembrane potential around the action potential) and **(c)** shows the injected current. Horizontal dashed line: 11.03 mV above the baseline for (a) and 523.7 mV/s for (b) corresponding to the threshold value estimated using the time derivative of the transmembrane potential. Threshold triggering the generation of an action potential was estimated to be 13.5 ± 2.8 mV for neurons (n = 9).

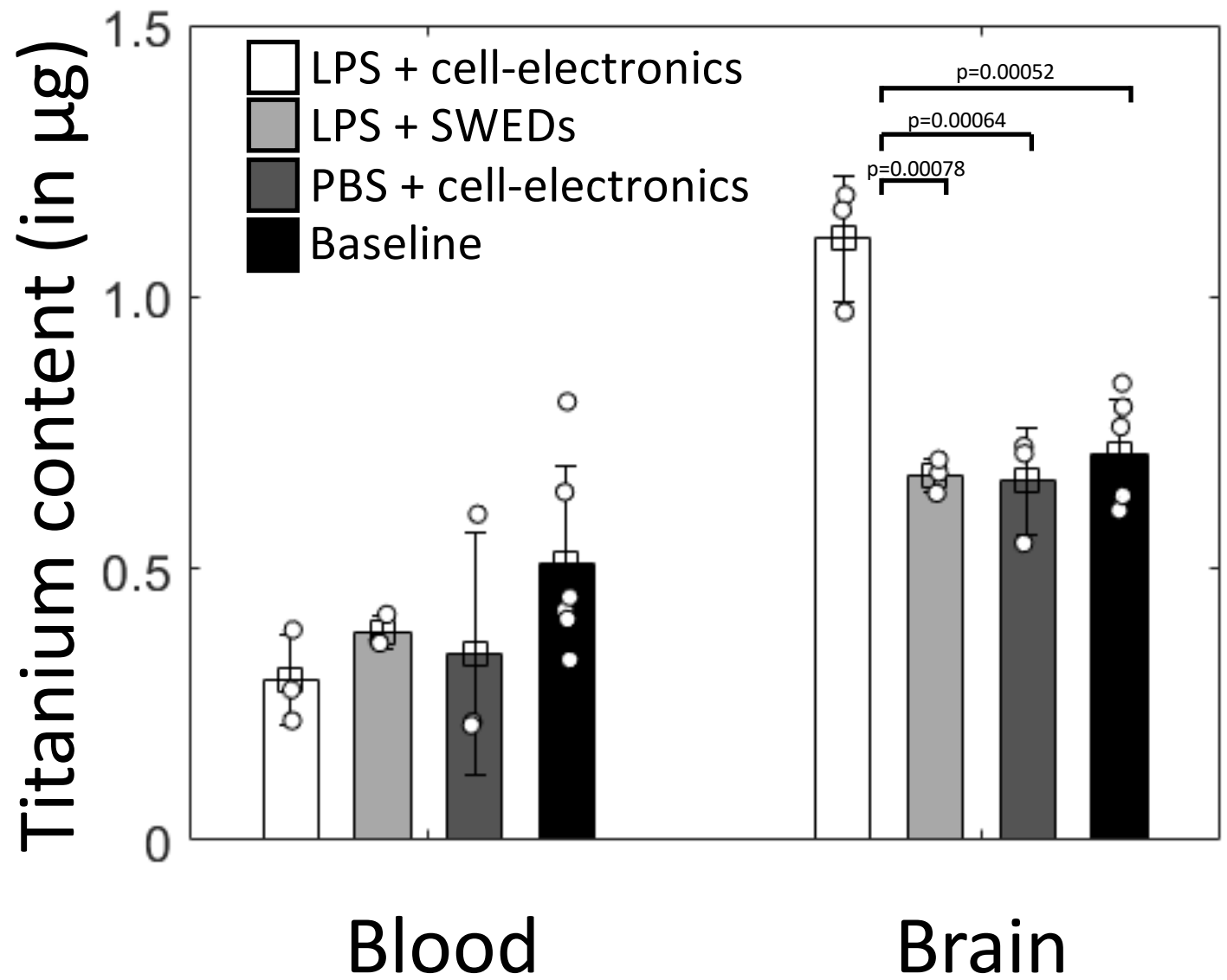

Supplementary Data Fig. S11. ICP-MS analysis

Titanium content as measured in the blood and the brain tissue for quantification of the SWEDs after three-day time-point post self-implantation (as SWEDs contain a 50 nm thin titanium layer) measured by Inductively Coupled Plasma Mass Spectrometry (ICP-MS) when 2 x 10^6 number of SWEDs only or cell-electronics hybrids were injected into the animal intravenously for the experimental and various control groups. A significant increase in titanium content compared to the baseline was observed only for the brain tissue in the experimental group while the changes in other control groups compared to baseline were insignificant, values represent mean ± SD (n = 3 mice), ****$p<0.0001$ (one-way ANOVA test).

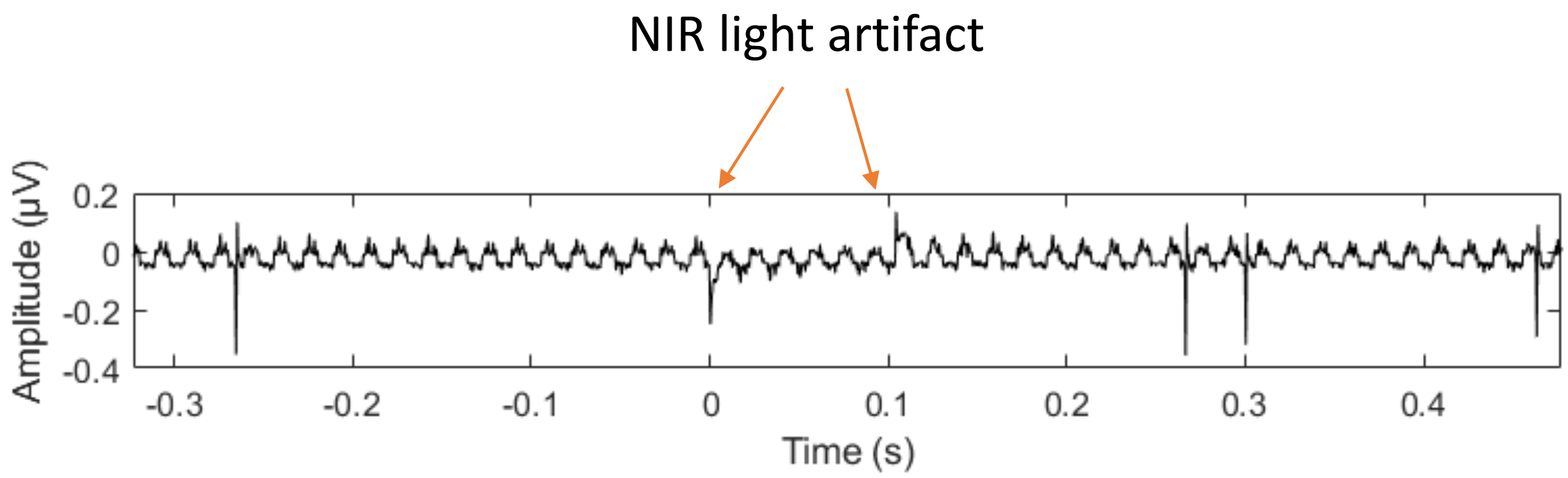

Supplementary Data Fig. S12: Multichannel single unit recording trace from a recorded unit showing a light artifact at the onset and the offset of the optical pulse.

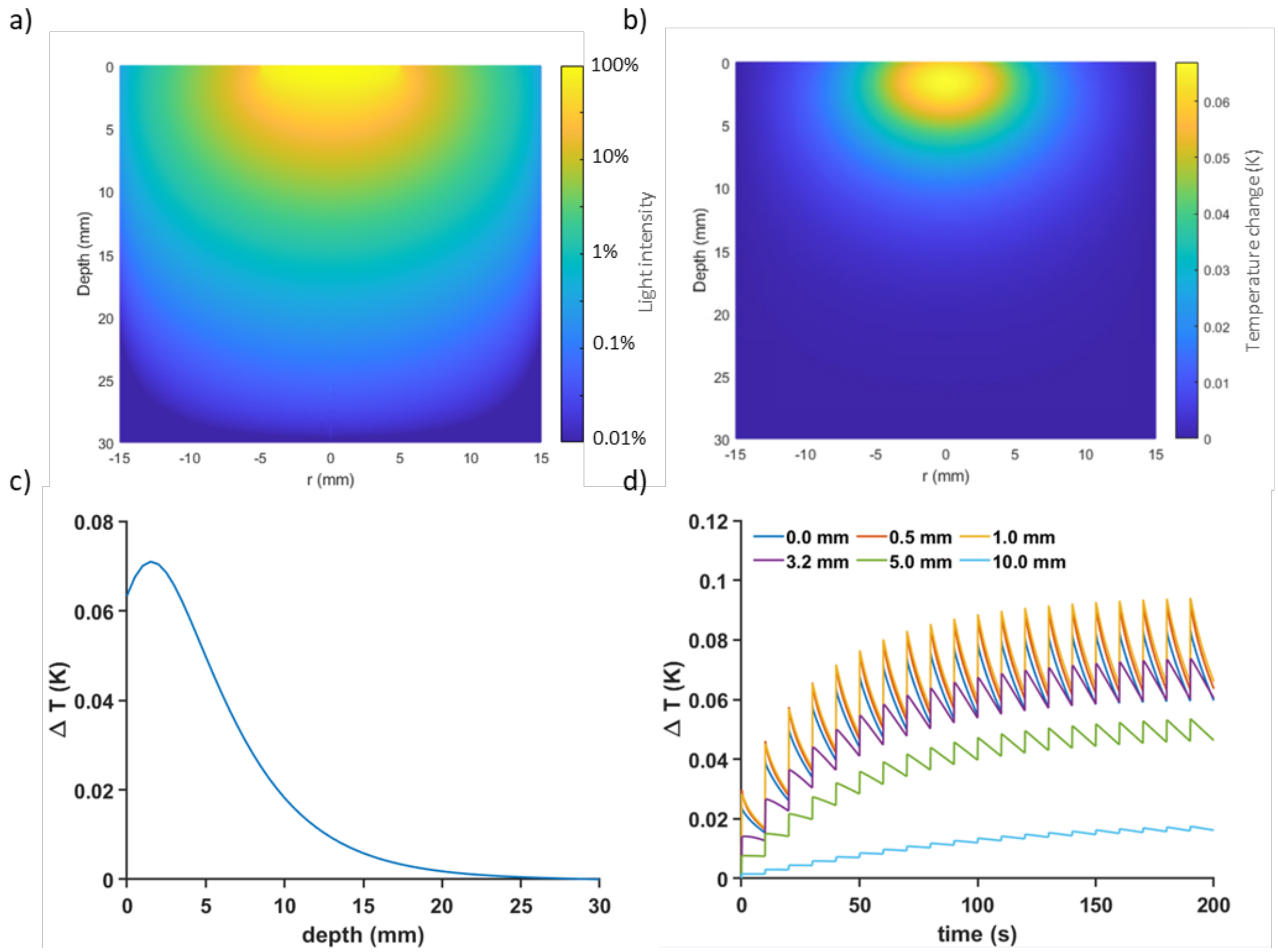

Supplementary Data Fig. S13: Monte Carlo modeling of light transport and heat diffusion study[3] in brain tissue. **(a)** Two-dimensional spatial distribution of light intensity with tissue depth. **(b)** Corresponding heat distribution map showing temperature elevation (in Kelvin) induced by light absorption throughout the tissue at t = 200s (when steady state is reached). **(c)** Temperature rise profile (at r = 0 mm, t = 200 s) as a function of tissue depth, illustrating the decay of thermal effects with increasing depth. **(d)** Temporal evolution of temperature changes (ΔT) at different tissue depths (0-10 mm), showing depth-dependent heating dynamics over 200 seconds of illumination for the optical pulse sequence used in this study. The simulation assumes collimated light incident at the tissue surface (depth = 0 mm). Optical pulse sequence – 100 ms pulse width, 0.1 Hz, 785 nm

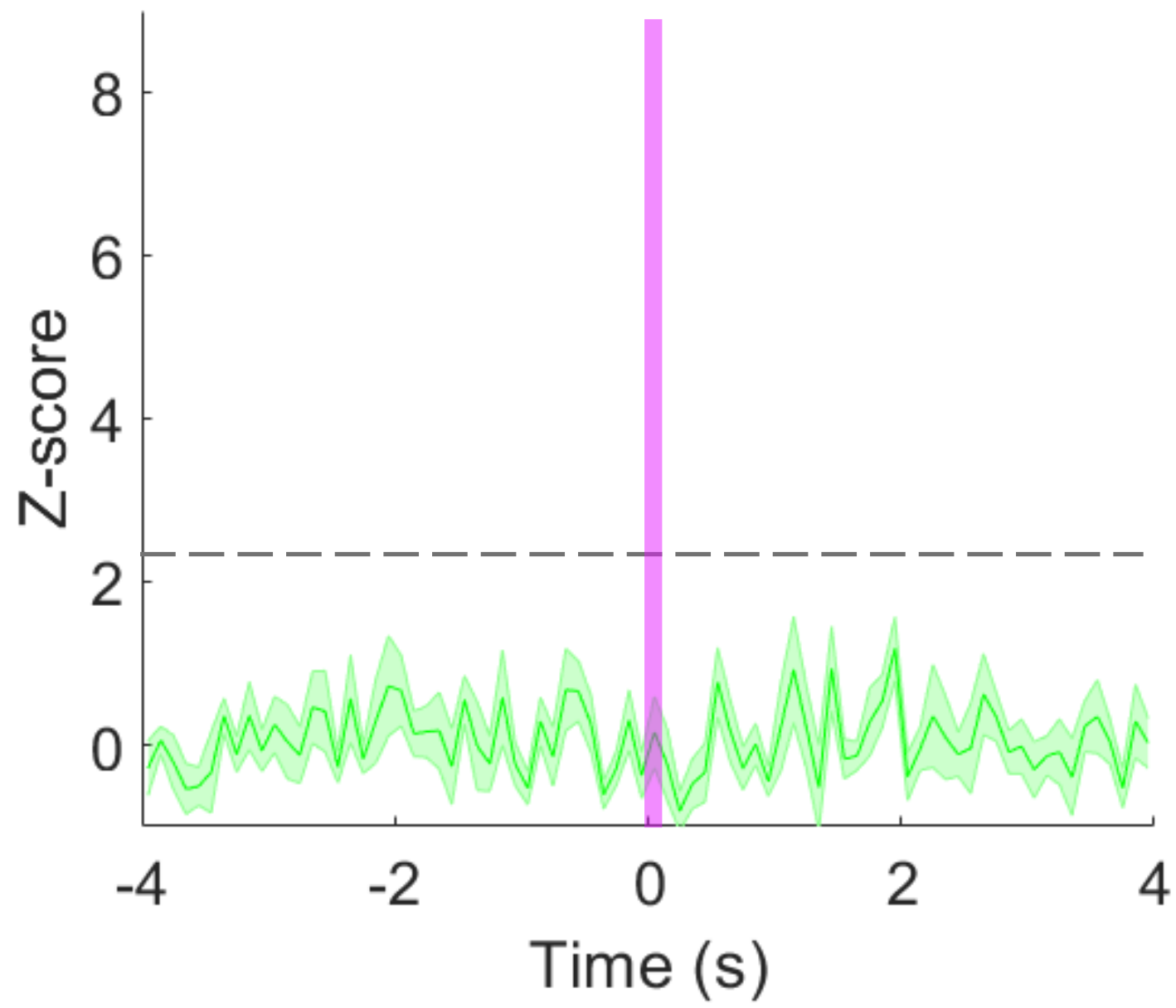

Supplementary Data Fig. S14: Normalized ensemble activities (z-scores) of non-active units from the experimental group (Hybrids + NIR Light, (n=50, 5mice)). Data are presented as mean ± SEM, horizontal dashed line – z=2.33, vertical shaded line - time = 0-100 ms for the optical pulse.

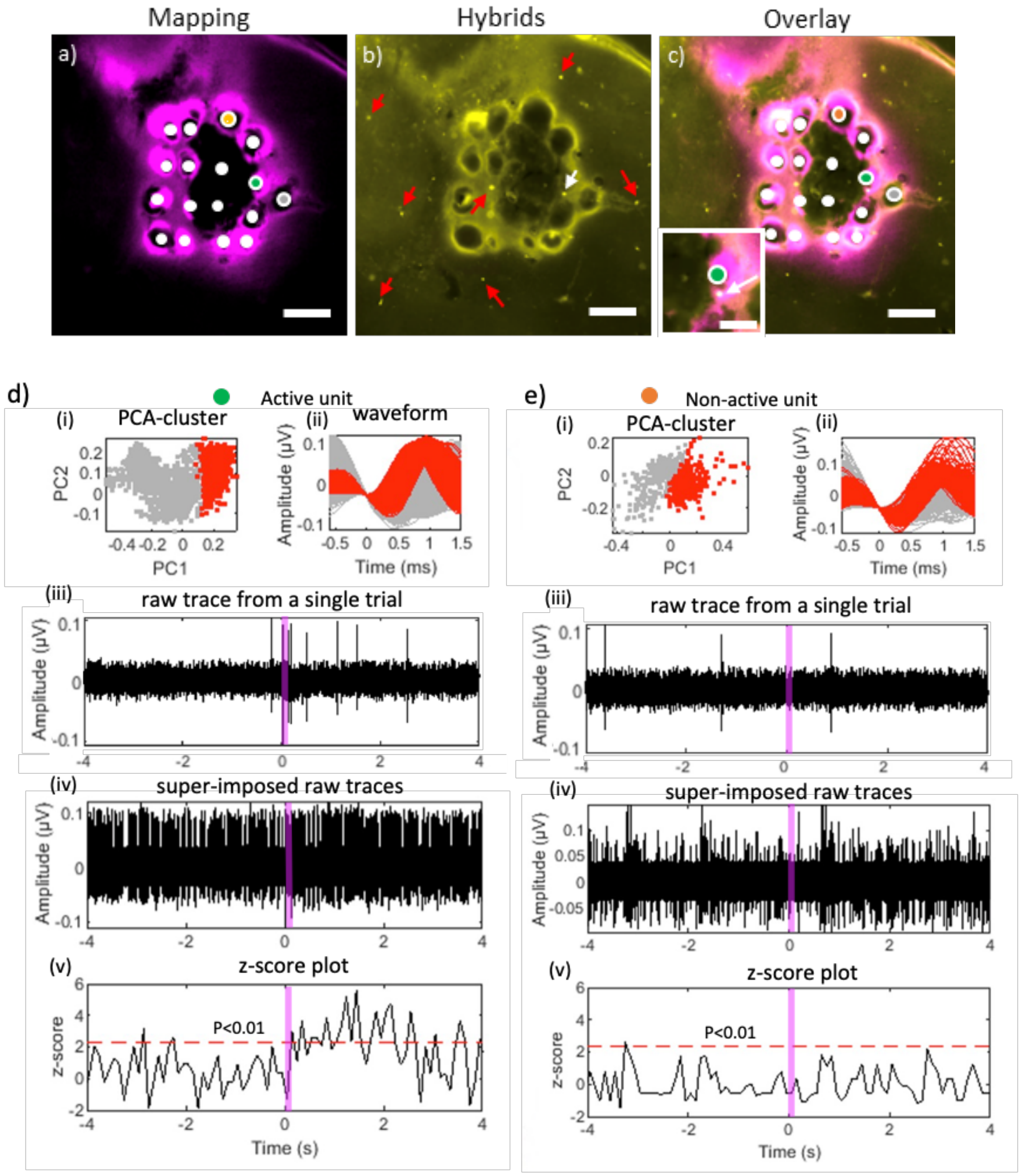

Supplementary Data Fig. S15: Fluorescent imaging for electrode mapping with hybrid localization. **(a)** DiI-stained electrode positions, **(b)** Fluorescent image of self-implanted hybrids (highlighted by arrows) and **(c)** overlay image (zoomed-in image showing the active unit with the hybrid right next to it). White – recording sites, grey – reference electrode, Scale bar: 250 µm (inset -100 µm), n = 2 for (a)-(c). (i) Principal component analysis (PCA) cluster, (ii) spike waveform, (iii) representative raw trace from a single trial, (iv) super-imposed raw-traces from multiple trials, and (v) z-score of the unit **(d)** as recorded by the electrode (highlighted in green) closer to the hybrid (indicated by the white arrow) and **(e)** as recorded by the electrode (highlighted in orange) farther away from the hybrids. Vertical shaded line ((iii)-(v)) - time = 0-100ms for the optical pulse. Horizontal dashed line (in z-score plot) – z=2.33, bin = 100ms, Optical pulse – 100 ms, 15 mW/mm^2, 792 nm.

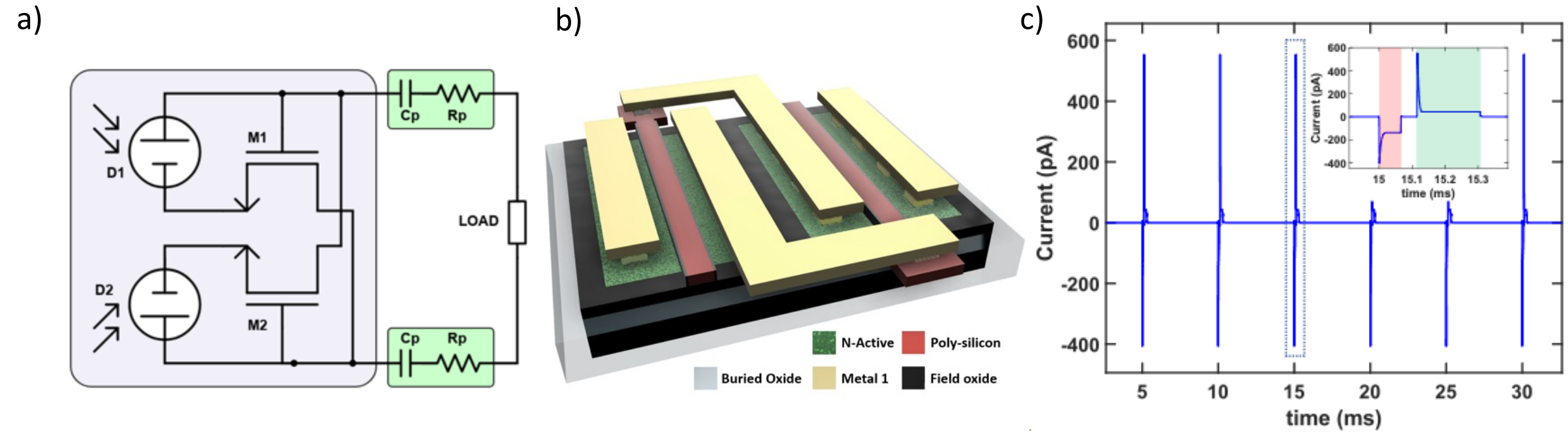

Supplementary Data Fig. S16. Towards integration of additional nanoelectronic circuits in Circulatronics. **(a)** Schematic circuit and **(b)** the layout for the proposed On-chip Biphasic pulse generator. Here, D_1, D_2 are P3HT:PCBM and PCPDTBT:PCBM based SWEDs; M_1, M_2 are MOSFETs modelled using BSIMSOI 4.7.1, and C_P, R_P are the pseudo-capacitance and parasitic-resistance modelled for the electrode-electrolyte interface. When D_1 (D_2) is turned ON, M_1 (M_2) turns ON and as a result, a positive (negative) current flows through the load. This circuit can be finely controlled for charge balance by tuning the ON/OFF time of the individual SWEDs. **(c)** Output waveform of the biphasic stimulator as obtained from simulations (for load = 1 kΩ). Inset: A single biphasic pulse, excited by a 785nm pulse (red) followed by 520nm pulse (green). SWED size – 10 µm in diameter for all the plots.

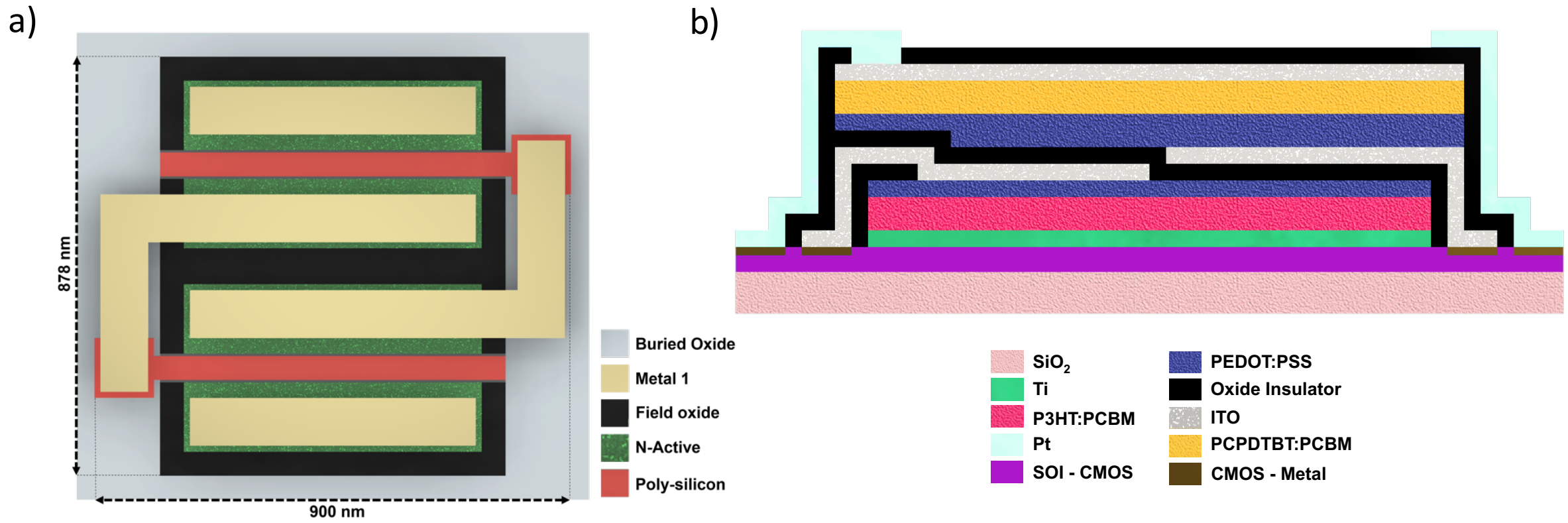

Supplementary Data Fig. S17. Layout of biphasic pulse generator

(a) Layout of the biphasic pulse generator circuit. The entire circuit can be implemented in less than 1 μm^2. **(b)** The schematic diagram of the biphasic pulse generator showing the individual layers. Here the CMOS circuit is shown as a bottom layer with SOI technology. Note that employing next-generation technologies such as 2D materials, electronic circuits can also be added as the top layers.

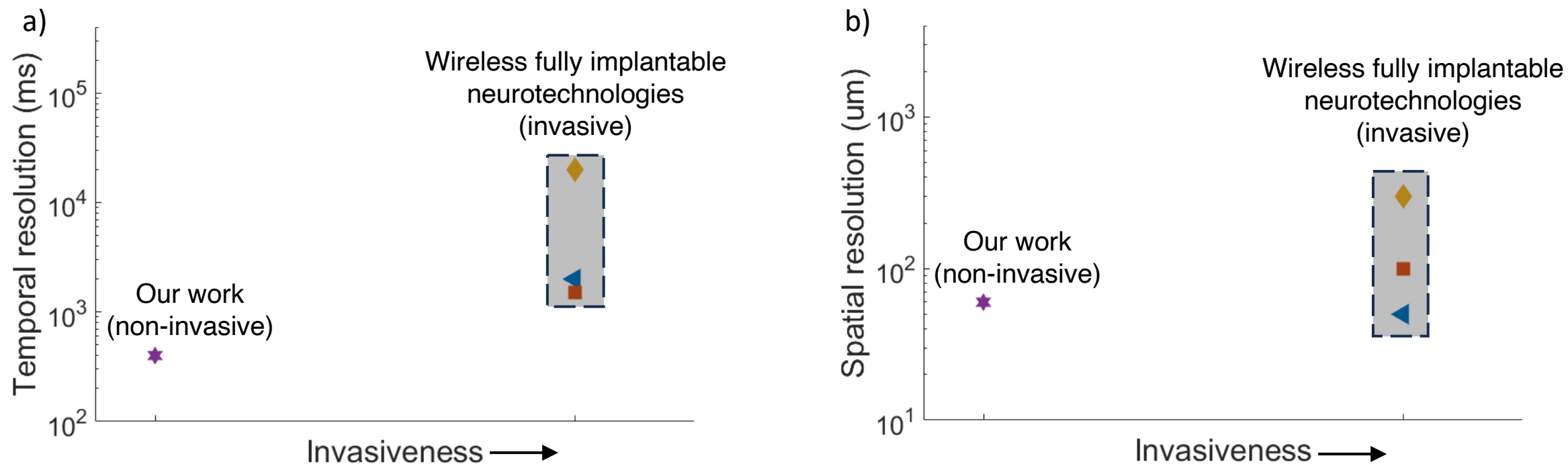

Supplementary Data Fig. S18: Figure showing **(a)** temporal and **(b)** spatial resolution of existing wireless fully implantable neurotechnologies for brain stimulation. Our technology demonstrates at par or better temporal response and spatial targeting compared to these technologies. In addition, this is the first demonstration of surgery-free and autonomous self-implantation of nanoelectronics at the target region to achieve focal neuromodulation. Ref: ◀[4], ■[5], and ◆[6].

Parameter	Units	Reference Range	Day 3, LPS Only	Day 3, Cell–Electronics Hybrids	Day 12, LPS Only	Day 12, Cell–Electronics Hybrids
WBC	K/µl	5.4–16.0	8.2 ± 1.31	6.16 ± 0.23	3.1 ± 1.41	3.85 ± 0.07
NEUT%	%	8.0–42.9	44.43 ± 6.86	46.4 ± 4.65	39.3 ± 0.98	39.25 ± 2.72
LYMPH%	%	55.0–95.0	45.63 ± 7.33	46.13 ± 5.35	52.4 ± 0.99	52.55 ± 7.71
MONO%	%	0.0–8.0	6.43 ± 2.55	5.73 ± 1.01	7.1 ± 0.56	7.25 ± 0.92
EOS%	%	0.0–2.9	2.86 ± 1.70	1.56 ± 0.35	1.15 ± 0.49	0.7 ± 0.56
BASO%	%	0.0–0.9	0.67 ± 0.35	0.2 ± 0.1	0.15 ± 0.07	0.2 ± 0.14
NEUT#	K/µl	—	3.67 ± 1.03	2.9 ± 0.26	1.2 ± 0.56	1.5 ± 0.28
LYMPH#	K/µl	—	3.7 ± 0.46	2.86 ± 0.37	1.65 ± 0.78	2 ± 0.28
MONO#	K/µl	—	0.57 ± 0.30	0.3 ± 0.057	0.2 ± 0.14	0.3 ± 0.0
EOS#	K/µl	—	0.23 ± 0.15	0.1 ± 0.0	0	0
BASO#	K/µl	—	0.067 ± 0.057	0	0	0
RBC	M/µl	6.70–12.50	10 ± 1.33	6.99 ± 0.58	8.87 ± 0.30	9.56 ± 0.15
HGB	g/dl	10.2–16.6	15.9 ± 2.1	10.83 ± 0.81	13.85 ± 0.92	14.9 ± 0.0
HCT	%	32.0–54.0	52.8 ± 7.02	36.6 ± 3.07	47.6 ± 2.3	50.15 ± 1.2
MCV	fl	31.0–62.0	52.8 ± 0.43	52.4 ± 0.1	53.8 ± 0.78	52.45 ± 0.35
MCH	pg	9.2–20.8	15.9 ± 0.26	15.53 ± 0.21	15.6 ± 0.49	15.6 ± 0.28
MCHC	g/dl	22.0–35.5	30.13 ± 0.32	29.6 ± 0.36	29.0 ± 0.49	29.7 ± 0.71
RDW	%	—	17.06 ± 0.50	16.7 ± 0.26	17.9 ± 0.71	19 ± 0.42
PLT	K/µl	150–2972	392.67 ± 266.7	822 ± 30.61	532.5 ± 55.2	591.5 ± 39.81
MPV	fl	—	5.6 ± 0.36	5.3 ± 0.26	5.35 ± 0.49	4.8 ± 0.14

Supplementary Table 1. Complete blood count analyses report of mice at 3 days and 12 days post LPS injection in the brain. Experimental group underwent administration of cell-electronics hybrids (2 million) via intravenous injection while control group did not, values represent mean ± SD (n=3 mice). All values were either in the normal reference range or no significant differences between the two groups were observed by unpaired two-tailed t-test for the 2 time-points.

Analyte	Unit	Reference Range	Day 3, LPS Only	Day 3, Cell–Electronics Hybrids	Day 12, LPS Only	Day 12, Cell–Electronics Hybrids
ALP	U/L	35–96	40.3 ± 11.59	39 ± 4	54.3 ± 6.65	67.3 ± 7.63
ALT	U/L	17–77	49.6 ± 3.51	35.3 ± 11.01	47.3 ± 29.1	43 ± 1
GGT	U/L	—	0	0	0	0
Amylase	U/L	—	518.3 ± 56.14	466.6 ± 59.8	777 ± 33	1167 ± 917.9
Albumin	g/dL	2.5–4.8	2.56 ± 0.45	2.63 ± 0.11	2.6 ± 0.26	2.6 ± 0.1
Total Bilirubin	mg/dL	0.1–0.9	0.2 ± 0.1	0.13 ± 0.057	0.13 ± 0.057	0.2 ± 0.0
Total Protein	g/dL	3.5–7.2	4.46 ± 0.61	4.56 ± 0.15	4.46 ± 0.305	4.46 ± 0.057
Globulin	g/dL	0.6–0.6	1.9 ± 0.173	1.93 ± 0.05	1.86 ± 0.057	1.86 ± 0.057
BUN	mg/dL	12.0–28.0	29.3 ± 12.09	27.3 ± 4.04	27.3 ± 4.16	23.3 ± 4.04
Creatinine	mg/dL	0.3–1	0.03 ± 0.057	0.1 ± 0.1	0.067 ± 0.05	0.03 ± 0.057
Cholesterol	mg/dL	29–110	81.6 ± 16.86	86.6 ± 12.05	112.67 ± 3.05	123.6 ± 8.9
Glucose	mg/dL	62–175	158.3 ± 34.78	141.3 ± 42.09	197 ± 34.2	198.3 ± 42.59
Calcium	mg/dL	4.8–10.1	8.86 ± 0.32	9.2 ± 0.435	8.53 ± 0.15	8.93 ± 0.057
Phosphorus	mg/dL	5.3–9.2	9.4 ± 1.91	7.26 ± 0.76	8.53 ± 1.36	8.73 ± 0.75
Chloride	mmol/L	88–129	110.5 ± 4.95	111.3 ± 1.53	112.33 ± 2.51	111.6 ± 2.5
Potassium	mmol/L	5–7.5	6.75 ± 1.62	6.53 ± 0.75	6.43 ± 0.49	6.86 ± 0.23
ALB/GLOB Ratio	—		1.3 ± 0.15	1.36 ± 0.057	1.36 ± 0.11	1.4 ± 0.1
Sodium	mmol/L	135–160	150.5 ± 2.12	152.6 ± 2.08	151.33 ± 2.39	149.6 ± 1.15

Supplementary Table 2. Blood serum analyses report of mice at 3 days and 12 days post LPS injection in the brain. Experimental group underwent administration of cell-electronics hybrids (2 million) via intravenous injection while control group did not, values represent mean ± SD (n=3 mice). All values were either in the normal reference range or no significant differences between the two groups were observed by unpaired two-tailed t-test for the 2 time-points.

Device	Volume (μm^3)	Device to biological cell volumetric ratio	Ability to circulate	Ability to enable non-surgical bioimplants	Ref
Device 1	1×10^8	$>10^5$	No	No	[7]
Device 2	8.1×10^9	$>10^7$	No	No	[8]
Device 3	4×10^7	$>10^5$	No	No	[9]
Device 4	7×10^6	$>10^4$	No	No	[10]
Device 5	1.7×10^8	$>10^5$	No	No	[11]
Device 6	6×10^8	$>10^6$	No	No	[12]
Device 7	5×10^7	$>10^5$	No	No	[13]
Device 8	1.7×10^8	$>10^5$	No	No	[14]
SWEDs	19.6	0.05	Yes	Yes	This work

Supplementary Table 3. Comparison of existing photovoltaic in vivo neuromodulation devices on their volumetric features, and their ability to freely move inside the vasculature and enable non-surgical bioimplants.

	**Eliminates invasive surgery**	**Autonomous recognition of Target**	**Focal electrical stimulation of eloquent structure**
Intracranial electrodes	No (intracranial surgery required)	No	Challenging (surgical placement poses risks of damage to eloquent structures)
Endovascular electrodes	No (endovascular surgery required)	No	No (steering endovascular electrodes to reach brain regions other than motor cortex is challenging)
Existing wireless devices	No (intracranial surgery/injection required)	No	No (surgical placement poses risks of damage to eloquent structures)
Existing non-invasive technologies (TMS, tDCS, tFUS)	Yes	No	No (TMS, tDCS – non-focal; tFUS –inhibition is heat induced; for excitation off-target brain activation occurs and functional isolation is rarely achieved)
**Circulatronics (Our work)**	Yes	Yes	Yes

Supplementary Table 4. Comparison of neural stimulation technologies highlighting key advantages of our work over existing approaches.

SUPPLEMENTARY NOTE

Comparison with existing neuromodulation technologies

Our technology has advantages compared to existing neuromodulation methods (**Supplementary Table 4**). Electrical stimulation based on wired electrodes are highly invasive and is only limited to about 1% of the patients[15]. Endovascular electrodes require surgical implantation, and are unable to access most of the brain regions[16-18]. Existing non-invasive stimulation techniques such as transcranial magnetic or electrical stimulation are unable to provide focal stimulation and face challenges due to non-consistent excitatory versus inhibitory stimulation[19] and techniques such as transcranial focused ultrasound (tFUS) provides heating-based inhibition and for excitation, tFUS leads to off-target brain activation and rarely achieves functional isolation[20,21]. Note that even wireless devices for neuromodulation also require invasive surgery including drilling of hole in the skull.

Our technology is not only wireless but also achieves spatial and temporal resolution comparable to or surpassing other wireless fully-implantable devices (**Supplementary Data Fig. S18**). Moreover, unlike existing approaches (be it wired or wireless), Circulatronics leverages the circulatory system as a global transport pathway, enabling intravenously introduced novel cell-electronics hybrid wherein the SWEDs are attached onto the cell surface—combining wireless optical energy harvesting (highest reported PCE) with cellular targeting capabilities—to circulate, autonomously navigate, recognize targets, and self-implant at the target brain regions without any external guidance or invasive surgery. To our knowledge, no other neuromodulation technology, regardless of modality, allows autonomous recognition and implantation of devices at the target region, eliminating the need for surgical intervention entirely.

Future Generations of Circulatronics

As the SWED demonstrated here is CMOS (BEOL) compatible and already includes a wireless power source (photovoltaic), they can be integrated with additional nanoelectronic circuitry for advanced functionalities. One such example is shown through simulations in **Supplementary Data Fig. S16** for generating charge-balanced biphasic pulses with user defined pulse-shapes. Generation of such biphasic pulses can be useful for biomedical applications such as chronic stimulation, for reducing adverse effect on tissue[22] as well as improving longevity of the bioelectronic interfaces[23]. The simulations are done employing the experimentally measured characteristics of our SWEDs (see **Methods 29**). This circuit involves two nanoscale Metal–Oxide–Semiconductor Field-Effect Transistors (MOSFETs) with 45 nm gate length and leverages the multiplexing capability and independent control of the P3HT and PCPDTBT based SWEDs to selectively switch these transistors to achieve biphasic pulses. The circuit layout is shown in **Supplementary Data Fig. S17a** which can easily fit in $< 1\ \mu m^2$. Cross sectional view of the pulse generator is shown in **Supplementary Data Fig. S17b. Supplementary Data Fig. S16c** shows the current waveform for the designed circuit with the successful generation of charge balanced biphasic pulses.

While the above circuit involves two transistors, employing 28 nm technology node and assuming a transistor footprint of approximately 0.1 μm in linear dimensions, integration of 1000s of transistors in a SWED (with 10 μm diameter) is possible. This number can be further increased by employing latest technology nodes[24] and 3D integration[25]. As biological events are slow (few milliseconds to hours), high performance (fast) transistors are not required for biomedical circuits and so, ultra-low-power transistors working in the subthreshold regime are ideal. For example, at a frequency of 1 kHz, such a transistor can be operated at less than 1pW of power[26,27]. The power generated by our highly efficient SWEDs when embedded in the tissue (**Fig. 2f-g**) can be used to operate 100s of such transistors in the whole rodent (mouse) brain with intact skull. Moreover, light transmission can be further improved to achieve penetration depths of several centimeters by using NIR laser source[1] as well as employing distributed optical emitter arrays[28]. Thus, light which also provides high spatio-temporal precision[29], can be a promising modality for powering implants inside the body and moreover, photo-biomodulation therapy has already been employed for clinical studies[30]. For example, employing NIR lasers (for improved light transmission) placed on human scalp and at incident averaged optical intensity of 100 mW/mm^2 on the scalp which is known to be safe, the power generated can be about 0.4 nW by the SWEDs when implanted in the brain (3 cm deep in

human head with intact skull having 2.9% transmission of light[1]) owing to their high power conversion efficiency (highest reported till date at such form factor, 4 orders of magnitude higher compared to previous reports[31]). This generated power can be used to operate many transistors in human brain with intact skull with each transistor having 1 pW of power requirement. This can be used to power electrical sensors or data analysis circuits for feedback-based operation. Note that while an optical power source has been demonstrated in the current work, other forms of energy harvesters (including radio-frequency electromagnetic, purely magnetic, acoustic) could be explored, based on the requirements of the specific applications.

The glorious growth of information technology is also accompanied by massive reduction in costs for mass production of nanoelectronics. The approximate cost for chips produced at a foundry is about US$ 10/cm^2. We could fit in about a million of subcellular sized electronic devices (10 μm in diameter) in 1 cm^2, with a cost of about US$ 0.00001 per device.

References:

1. Henderson, T. A. & Morries, L. D. Near-infrared photonic energy penetration: can infrared phototherapy effectively reach the human brain? *Neuropsychiatr. Dis. Treat.* **11**, 2191 (2015).
2. Salehpour, Farzad, et al. "Brain photobiomodulation therapy: a narrative review." Molecular neurobiology **55**, 6601-6636 (2018).
3. Stujenske, Joseph M., et al. "Modeling the spatiotemporal dynamics of light and heat propagation for in vivo optogenetics." *Cell reports* **12**, 525-534 (2015).
4. Chen, Keying, et al. "Potential of Photoelectric Stimulation with Ultrasmall Carbon Electrode on Neural Tissue: New Directions in Neurostimulation Technology Development." Advanced Functional Materials **34,** 2403164 (2024).
5. Kim, Ye Ji, et al. "Magnetoelectric nanodiscs enable wireless transgene-free neuromodulation." Nature Nanotechnology 1-11, (2024).
6. Kozielski, Kristen L., et al. "Nonresonant powering of injectable nanoelectrodes enables wireless deep brain stimulation in freely moving mice." Science advances **7**, eabc4189 (2021).
7. Silverå Ejneby, Malin, et al. "Chronic electrical stimulation of peripheral nerves via deep-red light transduced by an implanted organic photocapacitor." *Nature biomedical engineering* **6**, 741-753 (2022).
8. Gablech, Imrich, and Eric Daniel Głowacki. "State-of-the-Art Electronic Materials for Thin Films in Bioelectronics." *Advanced Electronic Materials.* **9**, 2300258 (2023).
9. Huang, Yunxiang, et al. "Bioresorbable thin-film silicon diodes for the optoelectronic excitation and inhibition of neural activities." *Nature Biomedical Engineering* **7**, 486-498 (2023).
10. Abdo, Ammar, et al. "Floating light-activated microelectrical stimulators tested in the rat spinal cord." *Journal of neural engineering* **8**, 056012 (2011).
11. Donahue, Mary J., et al. "Wireless optoelectronic devices for vagus nerve stimulation in mice." *Journal of neural engineering* **19**, 066031 (2022).
12. Sun, Pengcheng, et al. "A biodegradable and flexible neural interface for transdermal optoelectronic modulation and regeneration of peripheral nerves." *Nature Communications* **15**, 4721 (2024).
13. Prominski, Aleksander, et al. "Porosity-based heterojunctions enable leadless optoelectronic modulation of tissues." *Nature Materials* **21**, 647-655 (2022).
14. Li, P., Zhang, et al. “Monolithic silicon for high spatiotemporal translational photostimulation.” *Nature*, **626**, 990-998 (2024).
15. Sarica, C. et al. Trends and disparities in deep brain stimulation utilization in the United States: a Nationwide Inpatient Sample analysis from 1993 to 2017. Lancet Reg. Health Am. **26**, 100599 (2023).
16. Fan, J. Z., Lopez-Rivera, V. & Sheth, S. A. Over the Horizon: The Present and Future of Endovascular Neural Recording and Stimulation. *Frontiers in Neuroscience* **14**, 432 (2020).
17. Zhang, A. *et al.* Ultraflexible endovascular probes for brain recording through micrometer-scale vasculature. *Science* **381**, 306–312 (2023).
18. Opie, N. L. *et al.* Focal stimulation of the sheep motor cortex with a chronically implanted minimally invasive electrode array mounted on an endovascular stent. *Nat. Biomed. Eng.* **2**, 907–914 (2018).
19. Romero, Maria C., et al. "Neural effects of transcranial magnetic stimulation at the single-cell level." Nature communications, **10**, 2642 (2019).
20. Sato, Tomokazu, Mikhail G. Shapiro, and Doris Y. Tsao. "Ultrasonic neuromodulation causes widespread cortical activation via an indirect auditory mechanism." Neuron, **98**, 1031-1041 (2018).
21. Zhang, Tingting, et al. "Transcranial focused ultrasound neuromodulation: a review of the excitatory and inhibitory effects on brain activity in human and animals." Frontiers in human neuroscience **15**, 749162 (2021).
22. Merrill, D. R., Bikson, M. & Jefferys, J. G. R. Electrical stimulation of excitable tissue: design of efficacious and safe protocols. *J. Neurosci. Methods* **141**, 171–198 (2005).
23. Howell, B. & Grill, W. M. Design of electrodes for stimulation and recording. *Implant.*

Neuroprostheses Restoring Funct. 59–93 (2015).
24. NG Orji, YS Obeng, C Beitia, S Mashiro & J Moyne. International Roadmap for Devices and Systems. (2018).
25. Jiang, J., Parto, K., W Cao & Banerjee, K. Ultimate monolithic-3D integration with 2D materials: Rationale, prospects, and challenges. *IEEE J. Electron Devices Soc.* **7**, 878–887 (2019).
26. Vitale, S. A., Wyatt, P. W., N Checka, Kedzierski, J. & Keast, C. L. FDSOI process technology for subthreshold-operation ultralow-power electronics. *Proc. IEEE* **98**, 333–342 (2010).
27. Bryant, A. *et al.* Low-Power CMOS at Vdd=4kT/q. *Device Res. Conf. Conf. Dig. (Cat. No. 01TH8561)* 22–23 (2001).
28. Yue, L. & MS Humayun. Monte Carlo analysis of the enhanced transcranial penetration using distributed near-infrared emitter array. *J. Biomed. Opt.* **20**, 088001 (2015).
29. Ronzitti, E. *et al.* Recent advances in patterned photostimulation for optogenetics. *J. Opt.* **19**, 113001 (2017).
30. Salehpour, F. *et al.* Brain Photobiomodulation Therapy: a Narrative Review. *Mol. Neurobiol.* **55**, 6601–6636 (2018).
31. Parameswaran, R., Carvalho-de-Souza, J.L., Jiang, Y. et al. Photoelectrochemical modulation of neuronal activity with free-standing coaxial silicon nanowires. *Nature Nanotech* **13**, 260–266 (2018).
