# Supplementary material for: A nonsurgical brain implant enabled through a cell–electronics hybrid for focal neuromodulation
Source: Nat Biotechnol. 2025 Nov 5;44(8):1329–39. doi: 10.1038/s41587-025-02809-3 (PMC13461362; doi:10.1038/s41587-025-02809-3)
Supplement: Supplementary file 2 [file 41587_2025_2809_MOESM2_ESM.pdf]

nature portfolio

Corresponding author(s): Deblina Sarkar

Last updated by author(s): May 25, 2025

Reporting Summary

Nature Portfolio wishes to improve the reproducibility of the work that we publish. This form provides structure for consistency and transparency in reporting. For further information on Nature Portfolio policies, see our Editorial Policies and the Editorial Policy Checklist.

Statistics

For all statistical analyses, confirm that the following items are present in the figure legend, table legend, main text, or Methods section.

n/a	Confirmed	
☐	☒	The exact sample size (*n*) for each experimental group/condition, given as a discrete number and unit of measurement
☐	☒	A statement on whether measurements were taken from distinct samples or whether the same sample was measured repeatedly
☐	☒	The statistical test(s) used AND whether they are one- or two-sided<br>*Only common tests should be described solely by name; describe more complex techniques in the Methods section.*
☒	☐	A description of all covariates tested
☒	☐	A description of any assumptions or corrections, such as tests of normality and adjustment for multiple comparisons
☐	☒	A full description of the statistical parameters including central tendency (e.g. means) or other basic estimates (e.g. regression coefficient) AND variation (e.g. standard deviation) or associated estimates of uncertainty (e.g. confidence intervals)
☐	☒	For null hypothesis testing, the test statistic (e.g. *F*, *t*, *r*) with confidence intervals, effect sizes, degrees of freedom and *P* value noted<br>*Give P values as exact values whenever suitable.*
☒	☐	For Bayesian analysis, information on the choice of priors and Markov chain Monte Carlo settings
☒	☐	For hierarchical and complex designs, identification of the appropriate level for tests and full reporting of outcomes
☒	☐	Estimates of effect sizes (e.g. Cohen's *d*, Pearson's *r*), indicating how they were calculated

Our web collection on statistics for biologists contains articles on many of the points above.

Software and code

Policy information about availability of computer code

Data collection	Confocal images were collected using Nikon Ti microscope (CSU-X1 confocal module), Nikon 1AR Ultra-fast confocal microscope and confocal Olympus FV1200.<br>FIB/SEM images were taken using Zeiss Crossbeam 540 SEM/FIB,<br>IV characterization was done using CompactStat from Ivium Technologies,<br>Flow-Cytometry data was collected using Sony MA900-1.<br>ICP-MS data was collected using Agilent 7900.<br>Histology data was collected using TissueFAXS SL, Nikon 1AR Ultra-fast confocal microscope, Nikon Ti microscope (CSU-X1 confocal module).<br>MTT assay data was collected using Tecan platereader,<br>IVIS data was collected using IVIS Spectrum imaging equipment (PerkinElmer).<br>Behavioural experiment was collected using custom Matlab script and a CCD camera<br>Electrophysiology data was collected using DORIC Neuroscience studio/Openephys system and custom Matlab scripts<br>LTSpice XVII software was used for performing spice simulations<br>Transmigration study was carried out using a modified Boyden chamber kit (ECM558, Millipore Sigma) and EVOM2 (WPI) was used for measuring TEER
Data analysis	Matlab_R2023b, Microsoft Excel 16.80, Image J Fiji software 2.1, CytoSpec v11.8, NIS ELements AR v5.42, DragonFly version 2022.1.0.1249, Iviumsoft v4.1133, Cadence Virtuoso and Spectre, Cell Profiler v4.2.4, Living Image v4.7.2, QuPath v0.4.0, LTspice XVII

For manuscripts utilizing custom algorithms or software that are central to the research but not yet described in published literature, software must be made available to editors and reviewers. We strongly encourage code deposition in a community repository (e.g. GitHub). See the Nature Portfolio guidelines for submitting code & software for further information.

Data

Policy information about availability of data

All manuscripts must include a data availability statement. This statement should provide the following information, where applicable:

- Accession codes, unique identifiers, or web links for publicly available datasets
- A description of any restrictions on data availability
- For clinical datasets or third party data, please ensure that the statement adheres to our policy

Data availability: All data needed to evaluate the conclusions in the paper are present in the paper, the

Supplementary Materials, and/or the DRYAD repository51: DOI:10.5061/dryad.pzgmsbd12.

Code availability: Custom codes corresponding to different analysis can be found on the DRYAD

repository: DOI:10.5061/dryad.pzgmsbd12.

Research involving human participants, their data, or biological material

Policy information about studies with human participants or human data. See also policy information about sex, gender (identity/presentation), and sexual orientation and race, ethnicity and racism.

Reporting on sex and gender	NA
Reporting on race, ethnicity, or other socially relevant groupings	NA
Population characteristics	NA
Recruitment	NA
Ethics oversight	NA

Note that full information on the approval of the study protocol must also be provided in the manuscript.

Field-specific reporting

Please select the one below that is the best fit for your research. If you are not sure, read the appropriate sections before making your selection.

☒ Life sciences ☐ Behavioural & social sciences ☐ Ecological, evolutionary & environmental sciences

For a reference copy of the document with all sections, see nature.com/documents/nr-reporting-summary-flat.pdf

Life sciences study design

All studies must disclose on these points even when the disclosure is negative.

Sample size	We selected sample sizes to make the statistical power greater than 0.8.
Data exclusions	No data were excluded.
Replication	All experiments were replicated with the corresponding "n" mentioned in the manuscript
Randomization	Randomization was done for experiments as mentioned in the manuscript
Blinding	c-Fos positive cell counting and the histopathology analysis was done in a blinded fashion.

Reporting for specific materials, systems and methods

We require information from authors about some types of materials, experimental systems and methods used in many studies. Here, indicate whether each material, system or method listed is relevant to your study. If you are not sure if a list item applies to your research, read the appropriate section before selecting a response.

Materials & experimental systems

n/a	Involved in the study
☐	☒ Antibodies
☐	☒ Eukaryotic cell lines
☒	☐ Palaeontology and archaeology
☐	☒ Animals and other organisms
☒	☐ Clinical data
☒	☐ Dual use research of concern
☒	☐ Plants

Methods

n/a	Involved in the study
☒	☐ ChIP-seq
☐	☒ Flow cytometry
☒	☐ MRI-based neuroimaging

Antibodies

Antibodies used	Anti-c-Fos Rabbit, 1:500, ABE457, sigma<br>donkey anti-rabbit Alexa Fluor 647, 1:500, Thermofisher, A-31573<br>Hoechst 33342, 1:10000, Thermofisher, H3570<br>GFAP 2.2B10, 1:500, Thermofisher, 13-0300<br>IBA1 antibody, 1:500, SYSY, 234 308<br>Goat Anti-Rat IgG (H+L), Highly Cross-Adsorbed 488, 1:500, Biotium, 20023-1<br>Donkey Anti-Guinea Pig IgG (H+L), Highly Cross-Adsorbed 633, 1:500, Biotium, 20171-1
Validation	Antibodies used in this study were validated by the manufacturers.

Eukaryotic cell lines

Policy information about cell lines and Sex and Gender in Research

Cell line source(s)	ATCC: wehi265.1
Authentication	cells were authenticated by the manufacturers.
Mycoplasma contamination	All cell lines tested negative for mycoplasma contamination.
Commonly misidentified lines (See ICLAC register)	No commonly misidentified cell lines were used.

Animals and other research organisms

Policy information about studies involving animals; ARRIVE guidelines recommended for reporting animal research, and Sex and Gender in Research

Laboratory animals	Mice: BALB/c. 7–12 weeks old (Taconic). Female and male.<br>Mice were held in facilities with a 12-hr dark/light cycle. Air humidity was kept between 40%-70% in the facilities and average temperatures were held between 20-25 °C
Wild animals	The study did not involve wild animals.
Reporting on sex	Both male and female animals were used.
Field-collected samples	The study did not involve samples collected from the field.
Ethics oversight	All experiments were performed with the approval of the Institute Animal Care and use Committee of MIT

Note that full information on the approval of the study protocol must also be provided in the manuscript.

Plants

Seed stocks: NA

Novel plant genotypes: NA

Authentication: NA

Flow Cytometry

Plots

Confirm that:

☒ The axis labels state the marker and fluorochrome used (e.g. CD4-FITC).

☒ The axis scales are clearly visible. Include numbers along axes only for bottom left plot of group (a 'group' is an analysis of identical markers).

☒ All plots are contour plots with outliers or pseudocolor plots.

☒ A numerical value for number of cells or percentage (with statistics) is provided.

Methodology

Sample preparation: FlowCytometry was done as described. A single-cell suspension was created by filtering the solution from a 35 µm nylon mesh and stored on ice until sorting

Instrument: Sony MA-900-1 cell sorter

Software: Sony MA-900-1 cell sorter software and CytoSpec v11.8,

Cell population abundance: Post sort purity of cell-electronics hybrids was >90%

Gating strategy: Negative control of cells with no SWEDs was used to identify the baseline scattering and fluorescent signal. Cells with SWEDs attached to them showed higher scattering and fluorescence signal (SWEDs were functionalized with NHS-Cy3). This served as a double gating strategy that was employed to isolate the cell-electronics hybrids.

☒ Tick this box to confirm that a figure exemplifying the gating strategy is provided in the Supplementary Information.
